# Proline oxidase silencing inhibits p53-dependent apoptosis in MCF-7 breast cancer cells

**DOI:** 10.1007/s00726-021-03013-8

**Published:** 2021-06-04

**Authors:** Ilona Oscilowska, Thi Y. L. Huynh, Weronika Baszanowska, Izabela Prokop, Arkadiusz Surazynski, Mauro Galli, Piotr Zabielski, Jerzy Palka

**Affiliations:** 1 Department of Medicinal Chemistry, Medical University of Bialystok, Mickiewicza 2D, 15-222 Bialystok, Poland; 2 Department of Medical Biology, Medical University of Bialystok, Mickiewicza 2C, 15-222 Bialystok, Poland

**Keywords:** MCF-7 breast cancer cells, Proline oxidase, p53, Apoptosis, Proline dehydrogenase, Proline

## Abstract

Proline oxidase (POX) is mitochondrial proline-degrading enzyme of dual apoptosis/survival function. POX expression and proline availability are considered an underlying mechanism for differential POX functions. The mechanism for POX-dependent regulation of cell death/survival was studied in wild-type (MCF-7^WT^) and shRNA POX-silenced breast cancer cells (MCF-7^iPOX^). Proline concentration and proteomic analyses were determined by LC/MS/QTOF and LC/MS/ORBITRA, respectively. Inhibition of collagen biosynthesis (proline utilizing process) by 2-methoxyestradiol (2ME) contributed to induction of apoptosis in MCF-7^WT^ cells, as detected by increase in the expression of active caspase-3, -9 and p53. The process was not shown in MCF-7^iPOX^. In MCF-7^iPOX^ cells prolidase activity and expression as well as proline concentration were drastically increased, compared to MCF-7^WT^ cells. Down-regulation of p53 in MCF-7^iPOX^ cells was corroborated by proteomic analysis showing decrease in the expression of p53-related proteins. The mechanism for down-regulation of p53 expression in MCF-7^iPOX^ cells was found at the level of p53–PEPD complex formation that was counteracted by hydrogen peroxide treatment. In this study, we found that silencing POX modulate pro-survival phenotype of MCF-7 cells and suggest that the mechanism of this process undergoes through down-regulation of p53-dependent signaling.

**Supplementary Information:**

The online version contains supplementary material available at 10.1007/s00726-021-03013-8.

## Introduction

This report is continuation of studies on the role of proline oxidase (POX) in regulation of apoptosis/autophagy in MCF-7 cells with particular focus on relation between POX and p53. In our previous study, we found that POX silencing induced pro-survival phenotype in MCF-7 cells and proline availability played important role in this process (Zareba et al. 2017, 2018). It has been found that proline availability for POX-dependent functions is regulated by collagen biosynthesis, the main proline utilizing process (Zareba et al. 2017). Experimentally, 2-methoxyestradiol (2ME) was found as a potent inhibitor of collagen biosynthesis (Gelse et al. 2008; Jackson et al. 2020; Neamatallah et al. 2019; Salama et al. 2006) supporting proline for POX-dependent functions. However, the mechanism for interplay between POX and proline in regulation of apoptosis/autophagy is not well understood.

It is well documented that proline content in neoplastic cells is increased (Catchpole 2011; Hirayama 2009). However, the mechanism of proline accumulation in cells is not fully understood. Important source of proline may constitute collagen degradation products (Ii et al. 2006). Extracellular collagen degradation is initiated by metalloproteinases. It contributes to endocytosis of collagen degradation products and further degradation in lysosomes to free amino acids, except iminodipeptides, e.g., glycyl-proline. Iminodipeptides are degraded to amino acids in cytoplasm by specific iminodipeptidase, prolidase (PEPD). PEPD [E.C.3.4.13.9] also referred to peptidase D or imidopeptidase is a cytosolic imido-dipeptidase (Myara et al. 1984a; Palka and Phang 1997; Surazynski et al. 2008) that cleaves imido-dipeptides containing at C-terminal position proline or hydroxyproline (Mock and Green 1990). The physiologic substrate for PEPD is derived mainly from collagen degradation products and also from other degraded proline-containing proteins (Adibi and Mercer 1973; Jackson et al. 1975; Myara et al. 1984b). Collagen is rich in imido-bonds. In α1 subunit of type I procollagen (1464 amino acids), proline forms 119 bonds with glycine and in α2 subunit (1366 amino acids) such a doublet occurs 106 times. Most of proline is hydroxylated in matured collagen and un-hydroxylated proline in gly-pro doublet occurs 25 times (Jackson et al. 1975). PEPD releases proline from imido-dipeptides for collagen re-synthesis and therefore the enzyme plays an important role in turn-over of the protein.

Cytoplasmic localization of this enzyme is of great importance also in regulation of other proline-dependent metabolic responses (Palka and Phang 1997; Surazynski et al. 2008). Proline can be also utilized in mitochondria. This process is catalyzed by proline oxidase (POX).

POX, referred also to proline dehydrogenase (PRODH) is flavin-dependent enzyme associated with the inner mitochondrial membrane (Pandhare et al. 2009; Reiling and Sabatini 2006). It catalyzes the conversion of proline into ∆1-pyrroline-5-carboxylate (P5C). This reaction is important in maintaining the redox balance between mitochondria and cytoplasm. It seems that cytoplasmic proline that enters mitochondria is sensor of cellular energy status (Liu et al. 2010, 2012; Reiling and Sabatini 2006; Wise 2008). Free proline bearing reducing potential must be quickly utilized, producing FADH_2_. On the other hand, conversion of P5C to proline through NADPH/NADH is coupled to pentose phosphate pathway and glucose metabolism (Dang 2009; Pandhare et al. 2006; Reiling and Sabatini 2006; Wise et al. 2008).

Conversion of proline to P5C generates electrons that are transported to electron transport chain producing ATP for energy supply and survival (Liu et al. 2010, 2012; Reiling and Sabatini 2006) or they directly reduce oxygen, producing reactive oxygen species (ROS) that induce extrinsic or intrinsic apoptotic pathways (Dang 2009; Possemato 2011; Wang 2011; Wise et al. 2008). The mechanism that switches ATP/ROS generation is however unknown. Therefore, the identification of pathways involved in regulation of apoptosis/survival is of great importance. We suggest that p53 is involved in the mechanism of POX-dependent apoptosis/autophagy.

The most potent apoptosis-inducing factor is p53 (Zareba and Palka 2016; Zareba and Palka 2016; Zareba and Palka 2016; Zareba and Palka 2016; Zareba and Palka 2016; Zareba and Palka 2016; Zareba and Palka 2016). Protein p53 is known as a transcriptional activator of POX (Phang et al. 2008; Polyak et al. 1997) since POX promoter has a p53-response element (Maxwell and Kochevar 2008). Moreover, p53 activates PUMA protein, which promotes apoptosis by binding to and antagonizing anti-apoptotic Bcl-2 family members. Recently the link between PEPD and p53-dependent function was found. It has been documented that PEPD form complex with p53 regulating its tumor suppressing activity (Yang et al. 2017). Interestingly, this complex was found to be dissociated by hydrogen peroxide suggesting underlying mechanism for oxidative stress-induced p53-dependent apoptosis. In this study, we found that POX silencing in MCF-7 cells contributed to drastic decrease in p53 expression leading to pro-survival phenotype of the cells. The functional significance of this finding is discussed in this paper. The mechanism for POX-dependent regulation of cell death/survival was studied in wild-type (MCF-7^WT^) and shRNA POX-silenced breast cancer cells (MCF-7^iPOX^).

## Results

### Bullet points

Bullet points:

- Inhibition of collagen biosynthesis induces proline oxidase-dependent apoptosis in MCF-7 cells.
- Proline oxidase silencing induces pro-survival phenotype in MCF-7 cells
- Silencing of proline oxidase down-regulates p53 expression through complex formation with PEPD.

### The effect of proline availability on DNA biosynthesis, collagen biosynthesis, PEPD activity and intracellular proline concentration in MCF-7^WT^ and MCF-7^iPOX^ cells

The hypothesis that the intensity of proline catabolism by POX may represent an important mechanism by which cancer cells switch to apoptosis or survival mode led us to prepare MCF-7 cell line with stably silenced expression of POX (MCF-7^iPOX^), as described previously (Zareba et al. 2017) (Supplementary Material, SFig.1–SFig.5).

In order to evaluate the role of proline in apoptosis or survival in MCF-7^iPOX^ and MCF-7^WT^ breast cancer cells, we designed several experimental conditions to increase intracellular proline level by inhibiting collagen biosynthesis (proline utilizing process). 2-Methoxyestradiol (2ME), was used as an inhibitor of collagen biosynthesis (Gelse et al. 2008; Jackson et al. 2020; Neamatallah et al. 2019; Salama et al. 2006). Both MCF-7^WT^ and MCF-7^iPOX^ cells were cultured in growth medium without glutamine. 

**Fig. 1:**
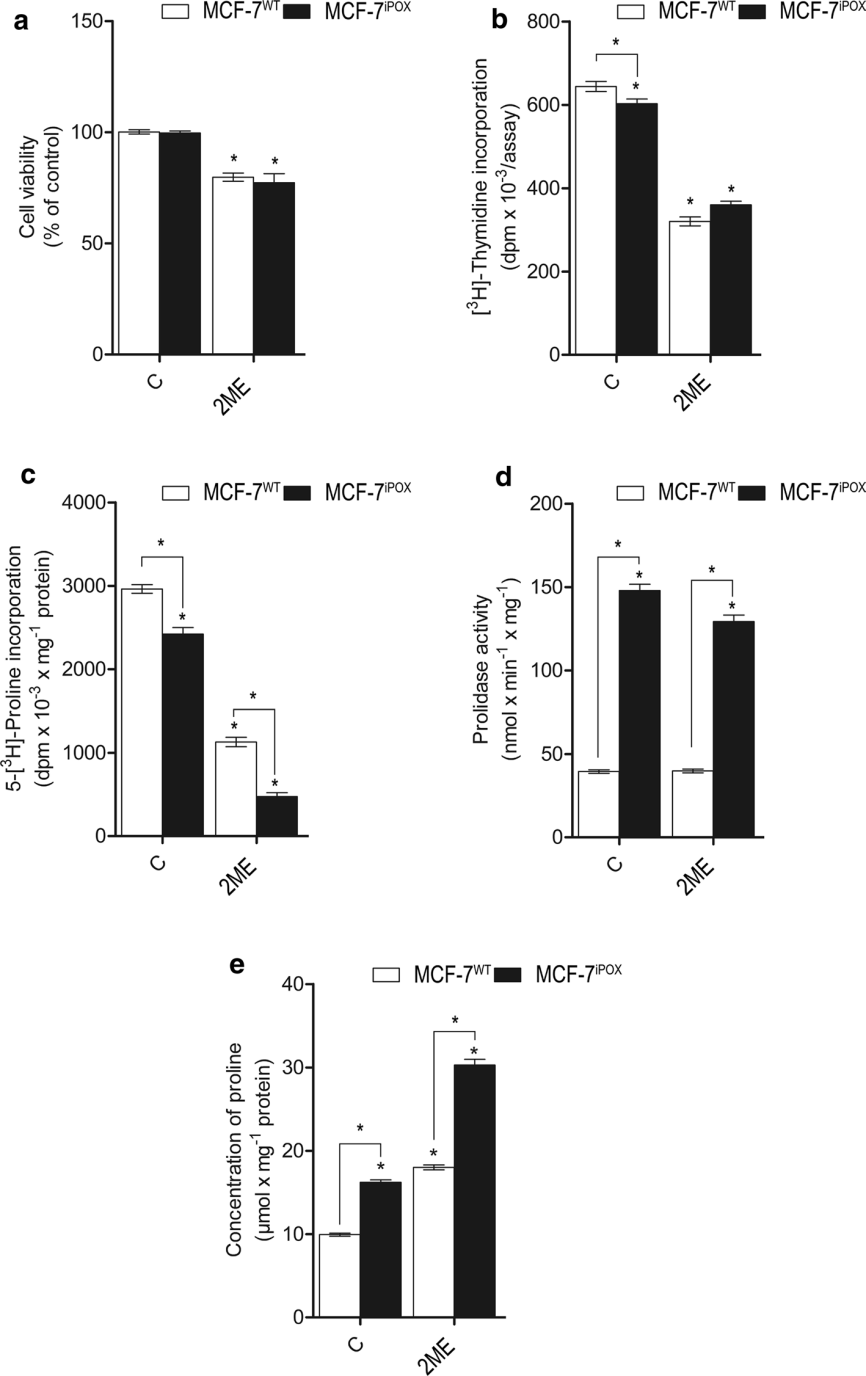
The effect of POX silencing and 2-methoxyestradiol (2ME) on processes that determine intracellular proline concentration and growth of breast cancer MCF-7 cells. Cell viability (**a**), biosynthesis of DNA (**b**) and collagen (**c**), prolidase (PEPD) activity (**d**) and intracellular proline concentration (**e**) in MCF-7^WT^ and MCF-7^iPOX^ cells cultured in DMEM without glutamine with (2ME) for 24 h. The mean values ± SD from 3 experiments done in duplicates are presented, *p < 0.01

As shown on Fig. 1a (time course experiment in Supplementary Material, SFig.6–SFig.9), POX silencing did not affect cell viability, however contributed to decrease in DNA (Fig. 1b, time course experiment in Supplementary Material, SFig.6–SFig.9) and collagen biosynthesis (Fig. 1c) and increase in prolidase activity (Fig. 1d) and intracellular proline concentration (Fig. 1e), compared to MCF-7^WT^ cells. When cellular proline level was increased by inhibition of proline utilization for collagen biosynthesis by 2ME (Fig. 1e), the cell viability, DNA and collagen biosynthesis were decreased in both MCF-7^iPOX^ and MCF-7^WT^ cells (Fig. 1a–c). Although 2ME maintained high prolidase activity only in MCF-7^iPOX^ cells (Fig. 1d), it contributed to increase in intracellular proline concentration in both cell lines (Fig. 1e).

It suggests that inhibition of collagen biosynthesis by 2ME or POX silencing increases intracellular proline concentration contributing to decrease in cell viability and DNA synthesis in MCF-7 cells.

### Down-regulation of POX induces pro-survival phenotype through p53–PEPD complex formation in MCF-7 cells

**Fig. 2:**
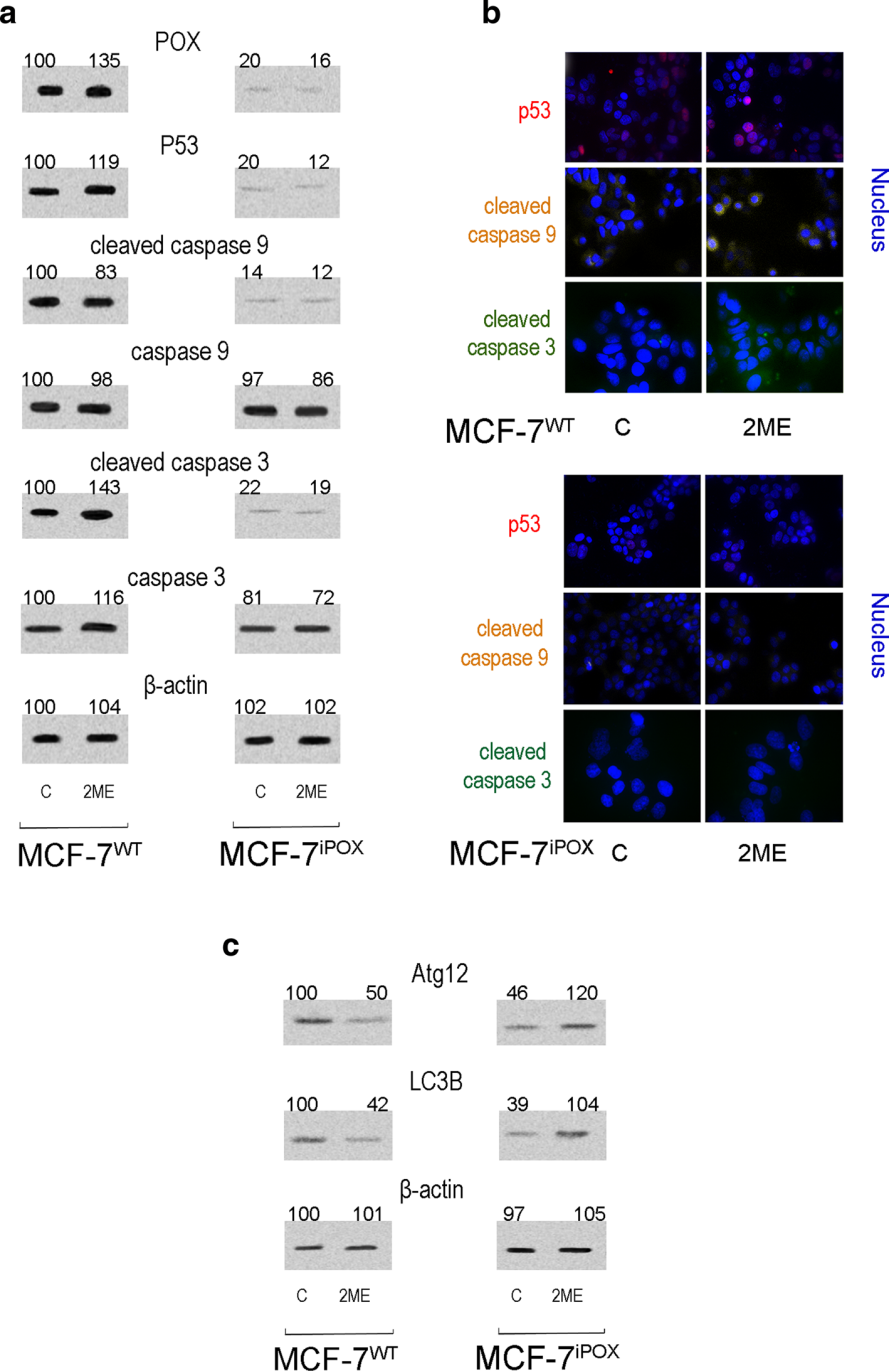
The effect of POX silencing and 2-methoxyestradiol (2ME) on apoptosis/autophagy in breast cancer MCF-7 cells. Western blot (**a**, **c**) and immunofluorescence bio-imaging (**b**) for p53, active and latent Caspases-3 and -9, Atg12 and LC3B in MCF-7^WT^ and MCF-7^iPOX^ cells cultured in DMEM without glutamine and submitted for 24 h to 2-methoxyestradiol (2ME). The WB bands intensity of representative gels was quantified by densitometry and normalized to β-actin (Supplementary Material, SFig.10–SFig.18). Bio-imaging pictures from confocal microscopy were taken at 200× (**b**). MCF-7^WT^ and MCF-7^iPOX^ cells were fixed and immunostained with appropriate antibody and Hoechst

Functional significance of POX silencing and proline availability for POX was found at the level of expression of p53, Caspase-3 and Caspase-9. As shown on Fig. 2 expression of p53 and active caspases -3 and -9 in MCF-7^iPOX^ cells was down regulated (Fig. 2a) compared to MCF-7^WT^ cells. In MCF-7^iPOX^ cells the effect was not affected by 2ME, while in MCF-7^WT^ cells 2ME induced POX, p53 and cleaved caspase-3 expressions. The results of this study were confirmed by immunofluorescence bio-imaging of p53 and active caspases -3 and-9 in MCF-7^iPOX^ cells and MCF-7^WT^ cells (Fig. 2b). In 2ME-treated MCF-7^WT^ cells all studied proteins were expressed, while in MCF-7^iPOX^ cells, the expression of these proteins was not detected. Instead, expression of autophagy markers (LC3B and ATG12) was found in this condition (Fig. 2c). In MCF-7^WT^ cells expression of Atg12 and LC3B was decreased upon 2ME treatment while in MCF-7^iPOX^ cells the expression of both autophagy markers was increased.

**Fig. 3:**
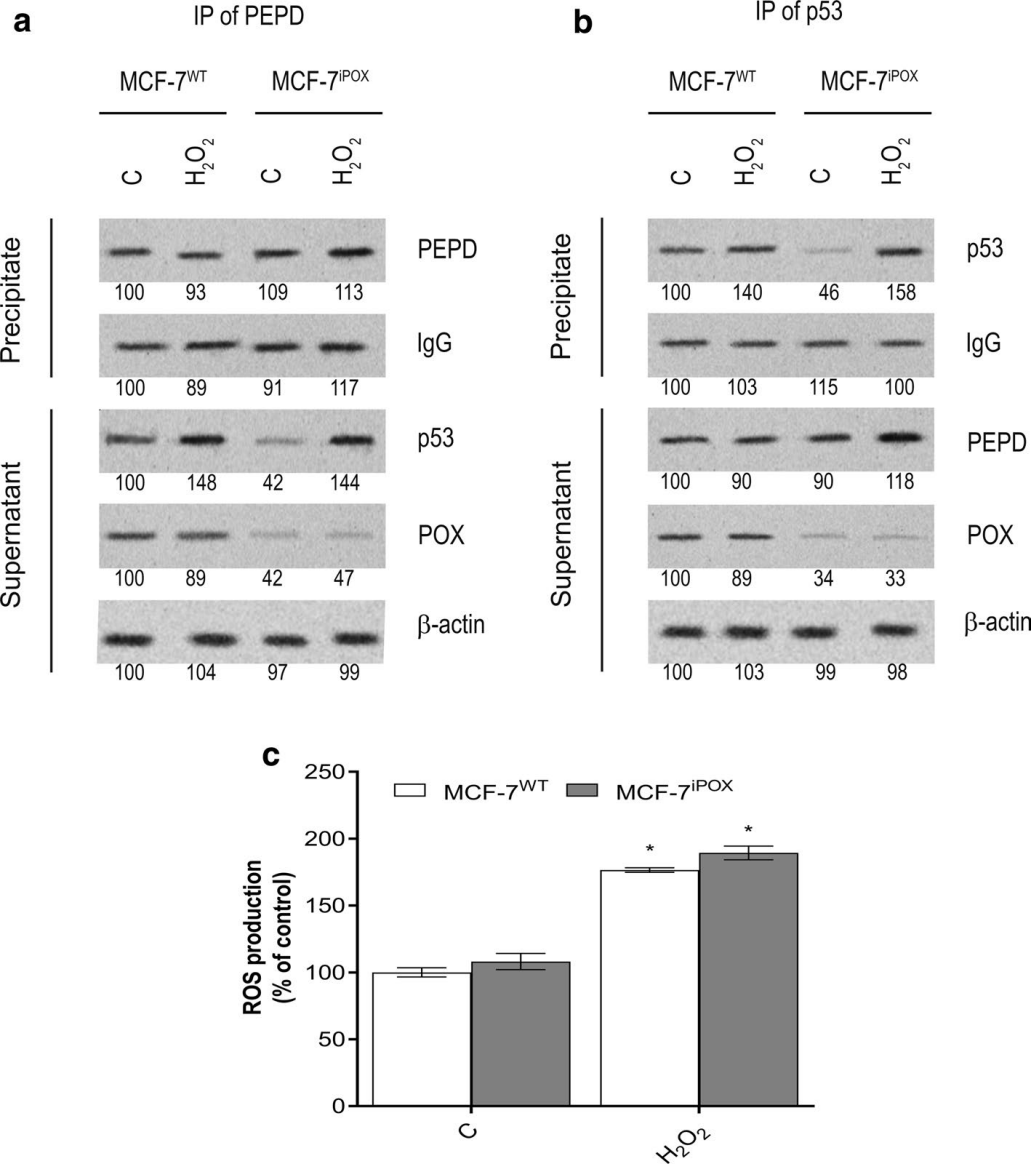
The effect of POX silencing on PEPD-p53 complex formation in breast cancer MCF-7 cells**.** Western blot for PEPD, p53 and POX after immunoprecipitation (IP) of PEPD (**a**) and p53 (**b**) in homogenates of MCF-7^WT^ cells and MCF-7^iPOX^ cells cultured in DMEM without glutamine and treated for 24 h with or without 400 µM hydrogen peroxide (H_2_O_2_). The WB bands intensity of representative gels was quantified by densitometry and normalized to IgG and β-actin, respectively. The densitometry values represent the mean (% of control) of three experiments (Supplementary Material, SFig.19–SFig.26). Validation of ROS generation by H_2_O_2_ treatment of the cells is presented on panel **c**

It suggests that POX silencing promotes pro-survival phenotype in MCF-7 cells. The potential mechanism for this process may involve inactivation of p53-dependent function by complex formation with PEPD (Yang et al. 2017). In fact, POX silencing induced dramatic increase in PEPD activity as presented in Fig. 1d, suggesting increase in the enzyme expression. Evidence for PEPD-p53 complex formation in POX-silenced cells was provided in 2 experiments in which PEPD and p53 were immunoprecipitated and in supernatants the presence of PEPD, p53 and POX were analyzed as presented in Fig. 3a, b. Low p53 expression in MCF-7^iPOX^ cells was recovered by 400 µM hydrogen peroxide. The phenomenon was not found in the MCF-7^WT^ cells.

The data suggest that elevation of prolidase in POX-silenced cells contribute to sequestration of p53 creating pro-survival phenotype of MCF-7 cells.

### POX silencing inhibits p53-regulated signaling in MCF-7 cells

**Fig. 4:**
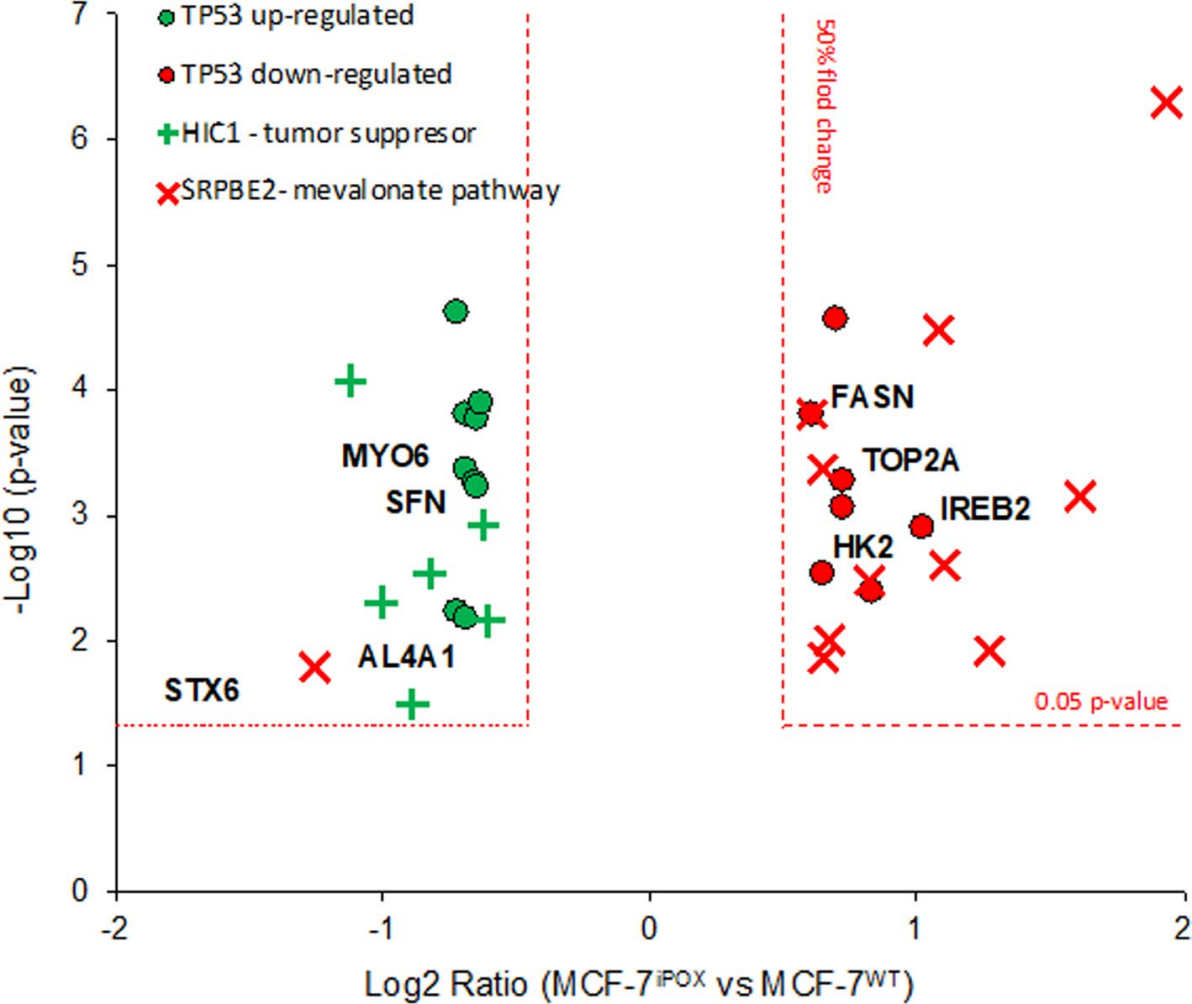
Expression of proteins known to be directly regulated by p53 activity, molecular members regulated by HIC1 (Hypermethylated In Cancer 1) and SREBF2 (Sterol Regulatory Element Binding Transcription Factor) involved in p53-dependent signaling. Volcano plot displays protein expression ratio between MCF-7^iPOX^ and MCF-7^WT^ cells expressed as Log2 fold change (MCF-7^iPOX^/MCF-7^WT^). Significance is expressed as − Log^10^ (p-value). Dashed red lines denote both the 50%-fold change cut-off (vertical lines with − 0.5 ≥ Log2 ratio ≥ 0.5, for down-regulation and up-regulation, respectively) and 0.05 *p*-value cut-off (horizontal lines, − Log10 (*p*-value) < 1.301 for *p*-value < 0.05). Proteins to the left of center are down regulated, whereas to the right of center up-regulated in MCF-7^iPOX^ as compared to MCF-7^WT^. Green circles and red circles represent proteins which are known to be directly up-regulated and down-regulated, respectively, by p53 activity. Green crosses represent proteins that are controlled by HIC1 transcription factor, whereas red crosses denote proteins controlled by SREBF2 transcription factor which belong to mevalonate pathway

To further elucidate the impact of POX inhibition and p53 down-regulation on the expression profile of proteins we performed proteomic analysis of MCF-7^iPOX^ and MCF-7^WT^ cells (Fig. 4). Out of 4414 identified proteins 416 displayed significantly different expression (down-regulation of up-regulation) between MCF-7^iPOX^ and MCF-7^WT^ cells. The dataset contained 16 proteins which are known to be directly regulated by p53 (Fig. 4) and 17 other which are members of p53-dependent pathways involved—both positively and negatively in the nodulation of the molecular function of p53. POX-silenced MCF-7 cells compared to wild-type MCF-7 displayed decreased expression of 9 proteins (e.g., STX6, ALDH4A1, RBBP4, MYO6, P4HB, GLUL, AKR1B1, SFN and LMAN2), which are known to be directly up-regulated by p53 at the level of transcription through the promoter response element. Among those STX6 [Syntaxin 6 (Zhang et al. 2008)], ALDH4A1 [Dehydrogenase Family 4 Member A1 (Yoon et al. 2004)], MYO6 [Myosin VI (Jung et al. 2006)] and SFN [Stratifin, 14-3-3 protein sigma (Yang et al. 2003)] are involved in p53-regulation of cell survival. Above data suggest that POX-mediated inhibition of p53 down-regulates proteins involved in negative control of cellular proliferation in p53-dependent manner. In line with above findings, we observed up-regulation of directly inhibited by p53 signaling (PPP3CA, TOP2A, HK2, IREB2, DLAT, ACSL3 and FASN) in MCF-7^iPOX^ cells as compared MCF-7^WT^. From listed above [IREB2 Iron regulatory protein 2 (Wang 2014b)], HK2 [Hexokinase 2 (Wang 2014a)], TOP2A [DNA topoisomerase 2-alpha (Yeo^ 2016^)] and FASN [fatty acids synthase, (Berkers et al. 2013)] play crucial role in cellular proliferation and transformation and their expression is up-regulated in p53-dependent cancers.

Figure 4 shows fold change [in log2 (ratio) format] and significance [in − log10 (*p*-value) format] of p53-regulated proteins, which were identified in proteomic dataset and proteins belonging to 2 major pathways revealed by upstream regulator analysis (URA) connected to p53 and involved in cell cycle regulation. In accordance with decreased expression of p53 in MCF-7^iPOX^ cells, we observed complementary response in the cellular level of p53-regulated proteins.

Upstream regulator analysis (URA) revealed through analysis of the expression profile of p53-dependent proteins overall inhibition of p53 signaling in MCF-7^iPOX^ as compared to MCF-7^WT^ with and overall *z*-score of − 1.106 and − Log10 (*p*-value) of 1.521, and (*p*-value of overlap of 6.20e-16). The above results suggest inhibition of p53-related signaling in MCF-7iPOX cells (Table 1). Moreover, URA revealed additional upstream regulators which are modulated by P53 activity. The proteins controlled by HIC1 (hypermethylated in cancer 1) transcription factor, that is widely regarded as a tumor suppressor gene, with p53 as its upstream regulator (Chen^ 2004^; Sun^ 2019^; Wales^ 1995^), also displayed down-regulation (*z*-score of − 1.951, − Log10 (*p*-value) of 1.673, *p*-value of overlap 7.12E-17, Table 1, Fig. 4). In line with the findings of decreased p53 signaling, we observed up regulation of p53-inhibited mevalonate pathway proteins, controlled by SREBF2 transcription factor (*z*-score of + 2.376, − Log10 (*p*-value) of 9.319, p-value of overlap < 3.21E-20). Mevalonate pathway—a source of substrates for sterol synthesis, protein isoprenylation and dolichol-linked n-glycosylation—is up-regulated in various cancers (including breast cancer) and p53 continuously suppresses its activity at the level of SREBF2 transcription factor (Moon^ 2019^; Yu 2021).

**Table 1:** Results of upstream regulator analysis (URA) and downstream effects analysis (DEA)

Upstream regulator analysis (URA) (MFC-7iPOX vs MCF7WT)					
Upstream regulator	Annotation	*z*-score	− log10 (*p*-val)	*p*-value of overlap	Target molecules
HIC1	Transcription regulator	− 1.951 ↓	1.673	7.12E-17	Table S2
SREBF2	Transcription regulator	+ 2.376 ↑	9.319	3.21E-20	Table S3

Downstream effects analysis (DEA)					
Category	Annotation	*z*-score	− log10 (*p*-val)	*p*-value of overlap	Target molecules
Cancer	Secondary tumor	− 1.377 ↓	3.087	8.18E-4	Table S2
Cancer	Breast cancer	− 0.849 ↓	4.170	6.76E-5	Table S3
Cancer	Mammary tumor	− 0.637 ↓	4.607	2.47E-5	Table S4

Table lists *z*-score values associated with the given transcription regulator (for URA) or downstream effects (for DEA), together with *p*-value of overlap [as − Log^10^ (*p*-value) and *p*-value, respectively]

↓ or ↑—predicted direction of change, down-regulation/up regulation (URA) or inhibition/activation or inhibition (DEA), respectively. URA allows for the identification of activated/inhibited upstream regulator, whereas DEA predicts downstream effects of the observed changes in protein expression. Detailed description of the statistics employed for the calculation of URA and DEA is included in the methods section

Downstream effects analysis (DEA) shows inhibition of cellular functions in MCF-7^iPOX^ cells connected to breast cancer (*z*-score = -0.849, − Log^10^*p*-value = 4.170, *p*-value < 6.76e^−5^), mammary tumor (*z*-score = − 0.637, − Log^10^*p*-value = 4.607, *p*-value < 2.47e^−5^) and secondary tumor and metastasis (*z*-score = − 1.377, − Log^10^*p*-value = 3.087, *p*-value < 8.18e^−4^). Table S1 to S4 lists molecular members identified by URA and DEA analysis from MCF-7^iPOX^ vs MCF-7^WT^ comparison, together with their expression Log^2^-ratio.

The data confirmed down-regulation of p53-dependent signaling in POX-silenced MCF-7 cells.

## Discussion

Here we provide further evidence for the role of POX expression and proline availability, as a substrate for this enzyme, in regulation of apoptosis/survival in MCF-7^WT^ cells. Availability of proline for degradation in mitochondria depends on the intensity of collagen biosynthesis that removes the amino acid from cytoplasmic pool limiting its conversion to P5C in mitochondria. We found that when collagen biosynthesis was inhibited, there was increase in cytoplasmic proline concentration and expression of pro-apoptotic markers. Similar effect was found previously in the cells treated with prolidase substrate, glycyl-proline (GP) that increased proline concentration in cytoplasm contributing to stimulation of collagen biosynthesis in MCF-7^WT^ cells and inhibition of this process in MCF-7^iPOX^ cells (Zareba et al. 2018). Although in MCF-7^iPOX^ cells cultured in the same conditions the viability, DNA and collagen biosynthesis were significantly decreased, there was no expression of active caspases-3 and -9. In fact, POX silencing in MCF-7 cells induced expression of autophagy markers and decreased DNA biosynthesis without any effect on the cell viability. Possibly, in the studied conditions the increase in the proline level in cytoplasm (occurred as a result of decreased POX expression and collagen biosynthesis) is not cytotoxic for the cells. However, deregulation of proline metabolism is known to deregulate DNA biosynthesis. Reducing potential of proline must be quickly converted in mitochondria to P5C by POX for regeneration of oxidizing potential. In cytoplasm, P5C is converted to proline by P5C reductase. The interconversion is known as a “proline cycle” (conversion of proline-P5C in mitochondria by POX and P5C-proline in cytosol by P5C reductase). It transfers reducing and oxidizing potential between mitochondria and cytosol using NAPDH/NADP + (Liu and Phang 2012; Liu et al. 2009). This shuttle is coupled to pentose phosphate shunt supporting biosynthesis of pyridine nucleotides (Liu et al. 2015). Therefore, deregulation of POX may affect DNA biosynthesis and cell proliferation.

Of special interest is observation that in MCF-7^iPOX^ cells collagen biosynthesis is decreased, while PEPD activity is increased. The mechanism of this process requires further studies. However, we hypothesize that the mechanism of collagen biosynthesis inhibition in MCF-7^iPOX^ cells may result from down-regulation of prolyl hydroxylase, an important enzyme in collagen biosynthesis. It has been documented that free proline inhibits prolyl hydroxylase (Surazynski et al. 2008; Zareba et al. 2017) suggesting a mechanism for proline-dependent attenuation of collagen biosynthesis. On the other hand, increase in PEPD activity in MCF-7^iPOX^ cells creates conditions for proline availability for POX-dependent functions. Therefore, we used 2ME, as an inhibitor of collagen biosynthesis to increase intracellular proline level for studying the effect on POX-dependent apoptosis and autophagy (Zareba et al. 2017). 2ME is a potent inhibitor of collagen biosynthesis (Gelse et al. 2008; Jackson et al. 2020; Neamatallah et al. 2019; Salama et al. 2006) supporting proline for POX-dependent functions (Zareba et al. 2017). In fact, previously we found that in such conditions there was increase in the expression of autophagy markers, as Atg7 and Beclin-1 (Zareba et al. 2017). The present results support the data showing increase in the expression of Atg12 and LC3B in 2ME-treated MCF-7^iPOX^ cells. Of special interest is that the differences between MCF-7^WT^ and MCF-7^iPOX^ cells with respect to apoptosis/autophagy phenotype were accompanied by differences in p53 expression. In MCF-7^iPOX^ cells the expression of p53 was drastically decreased.

Tumor suppressor p53 is known as the most potent inducer of POX activity (Phang et al. 2008; Polyak et al. 1997; Zareba et al. 2018). Transcriptional regulation of POX expression by p53 was found in the POX promoter, containing a p53-response element (Maxwell and Kochevar 2008). However, of great interest is the observation that p53 is down-regulated in MCF-7^iPOX^ cells.

In this study, we suggest that POX-dependent apoptosis in MCF-7^WT^ cells is mediated by p53, while POX silencing induces pro-survival phenotype in MCF-7 cells. The mechanism of this process cannot be explained on the basis of p53-dependent transcriptional regulation of POX since p53 is not regulated transcriptionally by POX (Kononczuk et al. 2015). The possible explanation for the process comes from recent report (Yang et al. 2017) showing that in human bladder cancer, human urothelial, murine myeloid 32D and human colon cancer cell lines, p53 can be suppressed by forming complex with PEPD. In fact, we found that in MCF-7^iPOX^ cells, PEPD activity was elevated providing conditions for sequestration of p53 and creation of pro-survival pathways. The supporting evidence for the p53–PEPD complex formation comes from experiment showing that in MCF-7^iPOX^ cells (and not in MCF-7^WT^ cells) hydrogen peroxide increased expression of both PEPD and p53. Recent studies documented that PEPD-p53 complex is dissociated by 400 µM hydrogen peroxide (Yang et al. 2017). In fact, in the presence of H_2_O_2_ the expressions of immunoprecipitated PEPD and p53 were increased in MCF-7^iPOX^ cell homogenates, compared to respective controls. It suggests that elevated amount of PEPD in MCF-7^iPOX^ cells facilitate p53 sequestration contributing to pro-survival phenotype of the cells.

It cannot be excluded that in studied conditions decrease in p53 expression could be partially due to the increase in proteasomal degradation of p53. P53 stability is augmented by hydroxylation of proline 359 residue of p53. It is supported by some studies (Rodriguez^ 2018^; Xu^ 2019^).

Since free proline (that is increased in studied conditions) inhibits prolyl hydroxylase (Surazynski et al. 2008), proline 359 residue of p53 is therefore not hydroxylated, loosing stability and could be directed for ubiquitination and proteasomal degradation.

The data suggest that expression of POX and PEPD as well as proline availability may regulate p53-dependent pro-apoptotic/pro-survival phenotype of MCF-7 cells.

The role of p53 in the mechanism of POX-dependent regulation of apoptosis in MCF-7^WT^ cells was supported by data from proteomic analysis. Down-regulation of p53 in MCF-7^iPOX^ cells promoting pro-survival phenotype was accompanied by several other p53-dependent pathways. Firstly, it has to be noted that down-regulation of p53 in MCF-7^iPOX^ cells led to simultaneous inhibition of proteins and pathways (e.g., HIC1, Fig. 4, Table 1) directly connected to p53-dependent regulation of cell survival. HIC1 transcription factor (involved in p53-dependent apoptotic DNA-damage response) acts as a tumor repressor, and its inhibition promotes pro-survival signaling and stimulates metastasis of breast cancer cells (Sun et al. 2019). Conversely, pro-survival and cancer connected pathways and proteins inhibited by p53 signaling, were up-regulated in MCF-7^iPOX^ cells. The SREBF2-regulated mevalonate pathway was one of the p53—inhibited pathways with highest positive *z*-score (Table 1), suggesting up-regulation due to absence of p53 inhibitory control. Mevalonate pathway—which is involved in biosynthesis of sterols and regulation of energy metabolism—evokes pro-survival signaling and is up-regulated in various cancers (Gong 2019; Guerra et al. 2021). The above results confirm that POX silencing in MCF-7^iPOX^ cells leads to down-regulation of p53 inhibitory control over cellular survival and up-regulation of pro-survival pathways. An example is drastic increase in SREBF2 expression, transcriptional activator of lipid metabolism that is often induced in response to starvation (Ivatt et al. 2014; Jiang et al. 2014; Moon et al. 2019; Wen et al. 2016). It suggests that POX silencing may affect utilization of energetic substrates leading to energy deficit. In fact, in MCF-7^iPOX^ cells a significant decrease in the expression of FASN (synthase of fatty acids) (Gaudet et al. 2011; Hu et al. 1996; Yoon et al. 2004) and HK2 (hexokinase 2) (Nawaz et al. 2018; Wang et al. 2014a) was found. It is well established that down-regulation of HK2 impairs glycolysis (Nawaz et al. 2018; Wang et al. 2014a) On the other hand, MCF-7^iPOX^ cells evoked high expression of ALDH4A1 (Gaudet et al. 2011; Hu et al. 1996; Srivastava et al. 2012), an enzyme catalyzing irreversible conversion of P5C (derived from proline or ornithine) into glutamic acid, supporting TCA cycle. It suggests that in conditions of POX silencing, deficiency of P5C and glutamate is compensated from urea cycle. The overall effect of reprogramming cellular metabolism in MCF-7^iPOX^ cells is activation of autophagy. It is supported by increase in the expression of STX6 (regulator of endosome trafficking) (Gaudet et al. 2011; Wang et al. 2005; Zhang et al. 2008), MYO6 (activator of ATPase) (Jung et al. 2006) and SFN (regulator of ubiquitination, cell growth and protein synthesis) (Chew et al. 2012; Leffers 1993; Metformin clinical trial; Samuel^ 2001^; Yang et al. 2008). Autophagy is also facilitated in MCF-7^iPOX^ cells by down-regulation of IREB2 (inhibitor of mRNA formation) (Gaudet et al. 2011; Khiroya^ 2017^; Samaniego et al. 1994) and TOP2A (positive regulator of apoptosis) (Wang et al. 1997; Yoshida et al. 2006).

Finally, we considered proline availability and POX as a molecular inter-face that can switch on and off survival or apoptotic mode. We found that up-regulation of proline concentration in cytoplasm by inhibition of its utilization for collagen biosynthesis by 2ME contributed to induction of apoptosis in MCF-7^WT^ cells, as detected by an increase in the expression of caspase-3 and -9, while in MCF-7^iPOX^ cells the process was inhibited and increase in the expression of autophagy markers was observed. Similar effect was observed previously where GP (substrate of prolidase) induced apoptosis in MCF-7WT cells as detected by an increase in the expression of caspase-3 and -9, while in MCF-7^iPOX^ cells increased expression of autophagy markers was observed (Zareba et al. 2018). The mechanism of POX-dependent regulation of apoptosis was found at the level of p53. In MCF-7^iPOX^ cells, this protein was not detected. Therefore, we suggest that POX silencing modulate pro-survival phenotype of MCF-7 cells through down-regulation of p53.

## Materials and methods

### Cell lines and culture

Breast cancer cell line MCF-7 (MCF-7^WT^) was obtained from ATCC (HTB-22, ATCC, Manassas, VA, USA). MCF-7^iPOX^ cell line was obtained by transfection of MCF-7^WT^ cells using plasmid with 3 different shRNA construct, which was described previously (Zareba et al. 2017, 2018). In this study, we used MCF-7^iPOX^ cells transfected by the most effective construct. More information is in the Additional file 1 of previous publication (Zareba et al. 2018). The MCF-7 and MCF-7^iPOX^ cells were maintained in DMEM (Gibco) and 10% FBS, 50 IU/ml penicillin and 50 μg/ml streptomycin in standard condition (37 °C in a humidified atmosphere containing 5% CO_2_). In the experimental conditions 80% confluent MCF-7^WT^ and MCF-7^iPOX^ cells were cultured in glutamine-free DMEM (in order to avoid proline generation from glutamine) and treated for 24 h with 2-methoxyestradiol (2ME, 72,7 μM) as an inhibitor of collagen biosynthesis.

### Western-immunoblot analysis

The procedure of Western-immunoblot analysis was described previously (Zareba et al. 2017, 2020). Protein concentration was measured by the method of Lowry et al. (Lowry et al. 1951) Cell lysates were subjected to SDS-PAGE in 10% polyacrylamide gel electrophoresis. After transfer, membranes were blocked with non-fat dry milk in TBS-T and incubated with goat anti-POX antibodies (Everest Biotech), rabbit anti-caspase-3 (Cell Signaling (CS)), rabbit anti-cleaved-caspase-3 (CS), rabbit anti-caspase-9 (CS), mouse anti-cleaved-caspase-9 (B&D), rabbit anti-PEPD (CS), rabbit anti-Atg12 (CS), rabbit anti-LC3B (CS) and mouse wild-type anti-p53 (B&D), mouse anti-β-actin (Sigma-Aldrich) diluted 1:1000 in blocking buffer. Then membranes were washed and incubated with respective HRP-linked secondary antibody at concentration 1:7500 (Sigma-Aldrich). Membranes were incubated with Amersham ECL Western Blotting Detection Reagent, (GE Healthcare Life Sciences). Pictures were taken using BioSpectrum Imaging System UVP (Ultra-Violet Products Ltd.

### Immunoprecipitation

Immunoprecipitation of proteins were done according to manufacturer’s procedure (Cell Signalling). 200 μg of cell lysate was incubated with primary antibody (PEPD and p53), then pre-washed magnetic-Sepharose with IgG beads were added. Next, the lysate, antibody and magnetic beads mix was incubated and rotated for 20 min. The supernatant was used to measure POX, PEPD and p53 (related to immunoprecipitation target) and β-actin expression. The pellet was resuspended and heated to 95–100 °C for 5 min. Then, by using magnetic rack, the mixture was separated. The supernatant was taken to Western blot analysis for p53, PEPD and IgG.

### Cell viability assay

The cell viability was determined using Nucleo Counter NC-3000 (ChemoMetec) as described before (Zareba et al. 2018). Prior the experiment MCF-7^WT^ and MCF-7^iPOX^ cells were cultured in six-well plates. After 24 h incubation of the cells in glutamine-free DMEM with or without 2ME cell viability assay was conducted according to company’s protocol.

### DNA biosynthesis assay

Proliferation of MCF-7^WT^ and MCF-7^iPOX^ cells was measured by [methyl-^3^H]thymidine (Hartman Analytic GmbH) incorporation into DNA. The DNA biosynthesis assay was performed as described previously (Zareba et al. 2017). Prior the experiment MCF-7^WT^ and MCF-7^iPOX^ cells were cultured in 24-well plate and treated for 24 h with or without 2ME in glutamine-free DMEM with 0.5 μCi/ml of [methyl-^3^H]thymidine. Incorporation of the tracer into DNA was measured by LiquidScintillation Analyzer Tri-Carb 2810 TR (Perkin Elmer) and calculated using QuantoSmart TM software (Perkin Elmer).

### Collagen biosynthesis

Incorporation of radioactive precursor into proteins was measured after the labeling of 80% confluent cells cultured in glutamine-free DMEM medium with 5[^3^H]-proline (5 μCi/ml) and with 2ME for 24 h. Incorporation of tracer into collagen was determined by digesting proteins with purified *Clostridium histolyticum* collagenase, accordance to the method of Peterkofsky et al. (Perekofsky et al. 1982). Results are shown as combined values for cell plus medium fractions. Incorporation of the tracer into collagen was measured by Liquid Scintillation Analyzer Tri-Carb 2810 TR (Perkin Elmer) and calculated using QuantoSmart TM software (Perkin Elmer).

### Determination of PEPD activity

The activity of PEPD was determined according to the method of Myara et al. (Myara et al. 1982). Protein concentration was measured by the method of Lowry et al. (Lowry et al. 1951). Enzyme activity was reported as nanomoles of proline released from synthetic substrate (GP), during 1 min per milligram of supernatant protein of cell homogenate.

### Immunofluorescence microscopy

Immunocytochemistry (ICC) was conducted according to BDB Bioimaging protocol, as described previously (Zareba et al. 2017). Cells grown on a coverslip were fixed with paraformaldehyde, then permeabilized with Triton and blocked with 3% FBS. Cells were incubated with primary antibodies (p53, caspase-3, cleaved-caspase-3 caspase-9, cleaved-caspase-9), subsequently with FITC Fluor-conjugated secondary antibody and Hoechst. Samples were visualized with a confocal laser scanning microscope (BD Pathway 855 Bioimager) using AttoVision software.

### ROS generation assessment

Intracellular reactive oxygen species accumulation was measured using DCFH-DA as a fluorescent probe. Briefly, cells were pre-incubated with DCFH-DA (20 µM) in culture medium for 30 min, washed twice with PBS and treated with H_2_O_2_. The fluorescent intensity was measured at excitation/emission wavelength of 488/535 nm using TECAN In-finite^®^ M200 PRO (Männedorf, Switzerland). The results were presented as a percent of the control value.

### Concentration of proline

The procedure of measurement intracellular proline concentration was described previously (Zareba et al. 2017, 2018). Proline level was measured by using HPLC system connected to QTOF (6530) mass spectrometry detector. As positive ionization mode used ESI. Samples were injected onto a HILIC column. Accurate mass measurements were obtained by online mass correction to reference masses delivered continuously during analyses; reference masses at m/z 121.0509 (protonated purine) and m/z 922.0098. The capillary voltage was set to 3000 V, the gas temperature was 330 °C, the nebulizer gas flow rate was 10,5 L/min. MS TOF parameters were as follows: fragmentor was set to 140 V, skimmer 65 V.

### Cellular proteome analysis

Cells from 8 independent experiments (MCF-7^WT^ and MCF-7^iPOX^, *n* = 4 per group) were washed twice with ice-cold PBS to remove cell culture medium and pelleted by centrifugation. Protein extraction and proteolytic digestion was performed according to Leon et al. (Leon et al. 2013) with minor modifications. Briefly, cells were suspended in ice-cold homogenization medium (5% sodium deoxycholate, 5 mM TCEP in 50 mM ammonium bicarbonate pH = 7.8) and sonicated on ice at 90% power for 60 s with the use of VCX 130 ultrasonic processor. Samples were reduced by heating to 60 °C for 30 min and alkylated with iodoacetamide (15 mM final concentration) for 30 min in room temperature in dark. After 10 × dilution with 50 mM ammonium bicarbonate samples were digested with Trypsin/LysC mix at 1:25 protein enzyme ratio for 12 h at 37 °C. After acidification with trifluoroacetic acid (final concentration 0.2%), sodium deoxycholate precipitate was removed by phase extraction with ethyl acetate. Samples were analyzed by LC/MS/MS on Thermo Dionex RS3500RSLC nanoflow chromatograph and Thermo Q-Exactive orbitrap. Briefly approx 1 μg of sample peptides were concentrated and desalted online using 300 µm i.d. × 5 mm Acclaim PepMap100 C18, 5 µm, 100 Å trap cartridge. Samples were resolved on 50 cm Acclaim PepMap RSLC C18, 2 μm, 100 Å, 75 μm I.D. column using 3-step, 4 h gradient of 0.1% formic acid in H_2_O as A and 80% acetonitrile/0.1% formic acid as B. Eluting peptides were analyzed by data dependent analysis (DDA) using the following settings: emitter voltage 2 kV (Nanospray Flex source with glass emitter and liquid junction) capillary temp. 275 °C, scan range 350–1750, MS1 resolution 70 K, ACG target 3e^6^ (minimum 6e^3^), maximum injection time 50 ms, MS2 resolution 17.5 K, ACG target of 2e^5^, maximum injection time 60 ms, isolation window of 3.0 m/z. Twenty most intense ions were selected for HCD fragmentation at 27 NCE. Previously fragmented precursors were excluded for 60 s. Isotopes of previously selected precursors and ions with unassigned charge, + 1 charge and > + 6 charge was not targeted for MS2. Resulting RAW files were uploaded to PEAKS 8.5 software and analyzed against human proteome database (Uniprot UP000005640) using decoy-based 0.5% FDR for peptide-spectrum matches, 2% for peptides and 5% for proteins. Carbamidomethylation was selected as fixed and deamidation of NQ and oxidation of M as variable PTM. Differential protein expression analysis was performed using PEAKS label-free internal statistics (ANOVA), with the following filtering: average area 1e^5^ counts, charge between 2 and 5, ID count of 3, detected in at least 4 samples, at least 1 unique peptide per protein, 1.5-fold (50% up- or down-regulation) expression difference and *p*-value of 0.05. Protein expression data was interpreted with Ingenuity Pathway Analysis Software (IPA) to identify altered molecular pathways and probable molecular regulators (Kramer et al. 2014; Qiagen). Analysis was performed with stringent filtering of pathways and relationships present in mammary gland, mammary gland tumors and breast cancer cell lines. Upstream regulator analysis (URA) and downstream effects analysis (DEA) *z*-score was performed with IPA internal statistics engine with threshold *p*-value set at 0.05 (Kramer et al. 2014).

### Statistical analysis

All experiments were analyzed using Prism 5 (GraphPad Software). All experiments were independently repeated at least three times. In experiments the mean values for six or three assays ± standard deviations (SD) were calculated. The results were submitted to statistical analysis using the Shapiro–Wilk test and Kolmogorov–Smirnov test. All results have a normal distribution. To assess statistical significance in conducted experiments, one-way ANOVA with Dunnett’s multiple comparison test with 99% confidence interval was used. Results were considered significant at *p < 0.01 level and are denoted by an asterisk (*). Significance of the results of proteomic analysis was estimated with the use of Ingenuity Pathway Analysis (IPA) software (Kramer et al. 2014; Qiagen). Briefly, the outcomes of the proteome differential expression analysis from Peak’s software were first uploaded into IPA system for core analysis and then overlaid with the global molecular network in the Ingenuity Pathway Knowledge Base (IPKB—evidence-based database). IPA internal statistics was used to identify canonical pathways, molecular regulators and molecular effects induced by POX silencing in MCF-7 cells. IPA Core Analysis was employed to calculate degree of change in biological pathways with right-tailed Fisher’s exact test. To identify the upstream regulators that can describe observed changes in protein expression or predict their biological effects we employed the IPA Upstream Regulator Analysis (URA), and IPA Downstream Effects Analysis (DEA), respectively. Both analysis use *z*-score statistics, which allows for prediction which transcription regulators are involved (e.g., p53, SREBF2, HIC1) and if they are activated or inhibited. The significance of *z*-score is calculated independently and expressed in − log10 (*p*-value), where *p*-value < 0.05 equals to − log10 (*p*-value) < 1.30103. Additionally, *p*-value of overlap was calculated with the use of Fisher’s exact test to further confirm whether there is a statistically significant overlap between the dataset proteins and the proteins that are regulated by a given TR.

## Conclusions

The studies were conducted to understand the mechanism of POX-dependent regulation of cell death and survival in breast cancer cells. We found that POX silencing modulates pro-survival phenotype of MCF-7 cells. The mechanism of this process undergoes through up-regulation of proline concentration and PEPD (proline-releasing enzyme), sequestration of p53 by PEPD and down-regulation of p53-dependent signaling. It explains the mechanism of pro-survival phenotype of MCF-7 cells and suggests that up-regulation of POX and down-regulation of PEPD may represent a novel strategy for breast cancer treatment.

## Patents

The sequences used to silence PRODH/POX expression were subject for patent application (patent application number: P.421954).

## Supplementary Information

Below is the link to the electronic supplementary material.

Supplementary file1 (DOCX 9048 KB)

Supplementary file2 (XLSX 20 KB)

## Data Availability

The datasets used and/or analyzed during the current study are available from the corresponding author on reasonable request.

## References

[CR1] Adibi SA, Mercer DW (1973). Protein digestion in human intestine as reflected in luminal, mucosal, and plasma amino acid concentrations after meals. J Clin Invest.

[CR2] Berkers CR, Maddocks OD, Cheung EC, Mor I, Vousden KH (2013). Metabolic regulation by p53 family members. Cell Metab.

[CR3] Catchpole G (2011). Metabolic profiling reveals key metabolic features of renal cell carcinoma. J Cell Mol Med.

[CR4] Chen W (2004). Epigenetic and genetic loss of Hic1 function accentuates the role of p53 in tumorigenesis. Cancer Cell.

[CR5] Chew YC, Adhikary G, Wilson GM, Xu W, Eckert RL (2012). Sulforaphane induction of p21(Cip1) cyclin-dependent kinase inhibitor expression requires p53 and Sp1 transcription factors and is p53-dependent. J Biol Chem.

[CR6] Dang CV (2009). MYC, microRNAs and glutamine addiction in cancers. Cell Cycle.

[CR7] Gaudet P, Livstone MS, Lewis SE, Thomas PD (2011). Phylogenetic-based propagation of functional annotations within the Gene Ontology consortium. Brief Bioinform.

[CR8] Gelse K, Pfander D, Obier S, Knaup KX, Wiesener M, Hennig FF, Swoboda B (2008). Role of hypoxia-inducible factor 1 alpha in the integrity of articular cartilage in murine knee joints. Arthritis Res Ther.

[CR9] Gong L (2019). The mevalonate coordinates energy input and cell proliferation. Cell Death Dis.

[CR10] Guerra B, Recio C, Aranda-Tavio H, Guerra-Rodriguez M, Garcia-Castellano JM, Fernandez-Perez L (2021). The mevalonate pathway, a metabolic target in cancer therapy. Front Oncol.

[CR11] Hirayama A (2009). Quantitative metabolome profiling of colon and stomach cancer microenvironment by capillary electrophoresis time-of-flight mass spectrometry. Cancer Res.

[CR12] Hu CA, Lin WW, Valle D (1996). Cloning, characterization, and expression of cDNAs encoding human delta 1-pyrroline-5-carboxylate dehydrogenase. J Biol Chem.

[CR13] Ii M, Yamamoto H, Adachi Y, Maruyama Y, Shinomura Y (2006) Role of matrix metalloproteinase-7 (matrilysin) in human cancer invasion, apoptosis, growth, and angiogenesis. Exp Biol Med 231 10.1177/153537020623100103 16380641

[CR14] Ivatt RM, Sanchez-Martinez A, Godena VK, Brown S, Ziviani E, Whitworth AJ (2014). Genome-wide RNAi screen identifies the Parkinson disease GWAS risk locus SREBF1 as a regulator of mitophagy. Proc Natl Acad Sci U S A.

[CR15] Jackson SH, Dennis AW, Greenberg M (1975). Iminodipeptiduria: a genetic defect in recycling collagen; a method for determining prolidase in erythrocytes. Can Med Assoc J.

[CR16] Jackson EK, Gillespie DG, Tofovic SP (2020). DPP4 inhibition, NPY1–36, PYY1–36, SDF-1alpha, and a hypertensive genetic background conspire to augment cell proliferation and collagen production: effects that are abolished by low concentrations of 2-methoxyestradiol. J Pharmacol Exp Ther.

[CR17] Jiang P, Gan M, Lin WL, Yen SH (2014). Nutrient deprivation induces alpha-synuclein aggregation through endoplasmic reticulum stress response and SREBP2 pathway. Front Aging Neurosci.

[CR18] Jung EJ, Liu G, Zhou W, Chen X (2006). Myosin VI is a mediator of the p53-dependent cell survival pathway. Mol Cell Biol.

[CR19] Khiroya H (2017). IRP2 as a potential modulator of cell proliferation, apoptosis and prognosis in nonsmall cell lung cancer. Eur Respir J.

[CR20] Kononczuk J, Czyzewska U, Moczydlowska J, Surażyński A, Palka J, Miltyk W (2015). Proline oxidase (POX) as a target for cancer therapy. Curr Drug Targets.

[CR21] Kramer A, Green J, Pollard J, Tugendreich S (2014). Causal analysis approaches in ingenuity pathway analysis. Bioinformatics.

[CR22] Leffers H (1993). Molecular cloning and expression of the transformation sensitive epithelial marker stratifin. A member of a protein family that has been involved in the protein kinase C signalling pathway. J Mol Biol.

[CR23] Leon IR, Schwammle V, Jensen ON, Sprenger RR (2013). Quantitative assessment of in-solution digestion efficiency identifies optimal protocols for unbiased protein analysis. Mol Cell Proteomics.

[CR24] Liu W, Phang JM (2012). Proline dehydrogenase (oxidase) in cancer. BioFactors.

[CR25] Liu Y, Borchert GL, Donald SP, Diwan BA, Anver M, Phang JM (2009). Proline oxidase functions as a mitochondrial tumor suppressor in human cancers. Cancer Res.

[CR26] Liu W (2010). miR-23b targets proline oxidase, a novel tumor suppressor protein in renal cancer. Oncogene.

[CR27] Liu W, Le A, Hancock C, Lane AN, Dang CV, Fan TW, Phang JM (2012). Reprogramming of proline and glutamine metabolism contributes to the proliferative and metabolic responses regulated by oncogenic transcription factor c-MYC. Proc Natl Acad Sci U S A.

[CR28] Liu W, Hancock CN, Fischer JW, Harman M, Phang JM (2015). Proline biosynthesis augments tumor cell growth and aerobic glycolysis: involvement of pyridine nucleotides. Sci Rep.

[CR29] Lowry OH, Rosebrough NJ, Farr AL, Randall RJ (1951). Protein measurement with the Folin phenol reagent. J Biol Chem.

[CR30] Maxwell SA, Kochevar GJ (2008). Identification of a p53-response element in the promoter of the proline oxidase gene. Biochem Biophys Res Commun.

[CR31] Metformin clinical trial. Accessed 30.04.2017 2017

[CR32] Mock WL, Green PC (1990). Mechanism and inhibition of prolidase. J Biol Chem.

[CR33] Moon SH (2019). p53 represses the mevalonate pathway to mediate tumor suppression. Cell.

[CR34] Myara I, Charpentier C, Lemonnier A (1982). Optimal conditions for prolidase assay by proline colorimetric determination: application to iminodipeptiduria. Clin Chim Acta.

[CR35] Myara I, Charpentier C, Lemonnier A (1984). Prolidase and prolidase deficiency. Life Sci.

[CR36] Myara I, Myara A, Mangeot M, Fabre M, Charpentier C, Lemonnier A (1984). Plasma prolidase activity: a possible index of collagen catabolism in chronic liver disease. Clin Chem.

[CR37] Nawaz MH, Ferreira JC, Nedyalkova L, Zhu H, Carrasco-Lopez C, Kirmizialtin S, Rabeh WM (2018). Biosci Rep.

[CR38] Neamatallah T, Abdel-Naim AB, Eid BG, Hasan A (2019). 2-Methoxyestradiol attenuates liver fibrosis in mice: implications for M2 macrophages. Naunyn Schmiedebergs Arch Pharmacol.

[CR39] Palka JA, Phang JM (1997). Prolidase activity in fibroblasts is regulated by interaction of extracellular matrix with cell surface integrin receptors. J Cell Biochem.

[CR40] Pandhare J, Cooper SK, Phang JM (2006). Proline oxidase, a proapoptotic gene, is induced by troglitazone: evidence for both peroxisome proliferator-activated receptor gamma-dependent and -independent mechanisms. J Biol Chem.

[CR41] Pandhare J, Donald SP, Cooper SK, Phang JM (2009). Regulation and function of proline oxidase under nutrient stress. J Cell Biochem.

[CR42] Perekofsky B, Chojkier M, Bateman J (1982) Determination of collagen synthesis in tissue and cell culture system. Immunochemistry of the extracellular matrix., Fufthmar M edn.,

[CR43] Phang JM, Pandhare J, Liu Y (2008). The metabolism of proline as microenvironmental stress substrate. J Nutr.

[CR44] Polyak K, Xia Y, Zweier JL, Kinzler KW, Vogelstein B (1997). A model for p53-induced apoptosis. Nature.

[CR45] Possemato R (2011). Functional genomics reveal that the serine synthesis pathway is essential in breast cancer. Nature.

[CR46] Qiagen https://www.qiagenbio-informatics.com/products/ingenuity-pathway-analys

[CR47] Reiling JH, Sabatini DM (2006). Stress and mTORture signaling. Oncogene.

[CR48] Rodriguez J (2018). PHD3 regulates p53 protein stability by hydroxylating proline 359. Cell Rep.

[CR49] Salama SA, Nasr AB, Dubey RK, Al-Hendy A (2006). Estrogen metabolite 2-methoxyestradiol induces apoptosis and inhibits cell proliferation and collagen production in rat and human leiomyoma cells: a potential medicinal treatment for uterine fibroids. J Soc Gynecol Investig.

[CR50] Samaniego F, Chin J, Iwai K, Rouault TA, Klausner RD (1994). Molecular characterization of a second iron-responsive element binding protein, iron regulatory protein 2. Structure, function, and post-translational regulation. J Biol Chem.

[CR51] Samuel T (2001). The G2/M regulator 14–3–3sigma prevents apoptosis through sequestration of Bax. J Biol Chem.

[CR52] Srivastava D, Singh RK, Moxley MA, Henzl MT, Becker DF, Tanner JJ (2012). The three-dimensional structural basis of type II hyperprolinemia. J Mol Biol.

[CR53] Sun X (2019). Tumor suppressor HIC1 is synergistically compromised by cancer-associated fibroblasts and tumor cells through the IL-6/pSTAT3 axis in breast cancer. BMC Cancer.

[CR54] Surazynski A, Donald SP, Cooper SK, Whiteside MA, Salnikow K, Liu Y, Phang JM (2008). Extracellular matrix and HIF-1 signaling: the role of prolidase. Int J Cancer.

[CR55] Wales MM (1995). p53 activates expression of HIC-1, a new candidate tumour suppressor gene on 17p13.3. Nat Med.

[CR56] Wang Q, Zambetti GP, Suttle DP (1997). Inhibition of DNA topoisomerase II alpha gene expression by the p53 tumor suppressor. Mol Cell Biol.

[CR57] Wang Y, Tai G, Lu L, Johannes L, Hong W, Tang BL (2005). Trans-Golgi network syntaxin 10 functions distinctly from syntaxins 6 and 16. Mol Membr Biol.

[CR58] Wang R (2011). The transcription factor Myc controls metabolic reprogramming upon T lymphocyte activation. Immunity.

[CR59] Wang L (2014). Hexokinase 2-mediated Warburg effect is required for PTEN- and p53-deficiency-driven prostate cancer growth. Cell Rep.

[CR60] Wang W (2014). IRP2 regulates breast tumor growth. Cancer Res.

[CR61] Wen G, Pachner LI, Gessner DK, Eder K, Ringseis R (2016). Sterol regulatory element-binding proteins are regulators of the sodium/iodide symporter in mammary epithelial cells. J Dairy Sci.

[CR62] Wise DR (2008). Myc regulates a transcriptional program that stimulates mitochondrial glutaminolysis and leads to glutamine addiction. Proc Natl Acad Sci U S A.

[CR63] Xu Y (2019). Prolyl hydroxylase 3 stabilizes the p53 tumor suppressor by inhibiting the p53-MDM2 interaction in a hydroxylase-independent manner. J Biol Chem.

[CR64] Yang HY, Wen YY, Chen CH, Lozano G, Lee MH (2003). 14–3–3 sigma positively regulates p53 and suppresses tumor growth. Mol Cell Biol.

[CR65] Yang W, Dicker DT, Chen J, El-Deiry WS (2008). CARPs enhance p53 turnover by degrading 14–3–3sigma and stabilizing MDM2. Cell Cycle.

[CR66] Yang L, Li Y, Bhattacharya A, Zhang Y (2017). PEPD is a pivotal regulator of p53 tumor suppressor. Nat Commun.

[CR67] Yeo CQX (2016). p53 maintains genomic stability by preventing interference between transcription and replication. Cell Rep.

[CR68] Yoon KA, Nakamura Y, Arakawa H (2004). Identification of ALDH4 as a p53-inducible gene and its protective role in cellular stresses. J Hum Genet.

[CR69] Yoshida K, Yamaguchi T, Shinagawa H, Taira N, Nakayama KI, Miki Y (2006). Protein kinase C delta activates topoisomerase IIalpha to induce apoptotic cell death in response to DNA damage. Mol Cell Biol.

[CR70] Yu R (2021). Mevalonate pathway inhibition slows breast cancer metastasis via reduced N-glycosylation abundance and branching. Cancer Res.

[CR71] Zareba I, Palka J (2016). Prolidase-proline dehydrogenase/proline oxidase-collagen biosynthesis axis as a potential interface of apoptosis/autophagy. BioFactors.

[CR72] Zareba I, Surazynski A, Chrusciel M, Miltyk W, Doroszko M, Rahman N, Palka J (2017). Functional consequences of intracellular proline levels manipulation affecting PRODH/POX-dependent pro-apoptotic pathways in a novel in vitro cell culture model. Cell Physiol Biochem.

[CR73] Zareba I, Celinska-Janowicz K, Surazynski A, Miltyk W, Palka J (2018). Proline oxidase silencing induces proline-dependent pro-survival pathways in MCF-7 cells. Oncotarget.

[CR74] Zareba I (2020). Overexpression of prolidase induces autophagic death in MCF-7 breast cancer cells. Cell Physiol Biochem.

[CR75] Zhang Y, Shu L, Chen X (2008). Syntaxin 6, a regulator of the protein trafficking machinery and a target of the p53 family, is required for cell adhesion and survival. J Biol Chem.

