# Supplementary material for: Proline oxidase silencing inhibits p53-dependent apoptosis in MCF-7 breast cancer cells
Source: Amino Acids. 2021 Jun 4;53(12):1943–56. doi: 10.1007/s00726-021-03013-8 (PMC8651586; doi:10.1007/s00726-021-03013-8)
Supplement: Supplementary file 1 — Supplementary file1 (DOCX 9048 KB) [file 726_2021_3013_MOESM1_ESM.docx]

Amino Acids

Supplementary Material

Proline Oxidase silencing inhibits p53-dependent apoptosis in MCF-7 breast cancer cells

Ilona Oscilowska^1^, Thi YL Huynh^1^, Weronika Baszanowska^1^, Izabela Prokop^1^, Arkadiusz Surazynski^1^, Mauro Galli^2^, Piotr Zabielski^2^ and Jerzy Palka^1,^*

^1^ Department of Medicinal Chemistry, Medical University of Bialystok, Mickiewicza 2D, 15-222 Bialystok, Poland; e-mail: pal@umb.edu.pl

^2^ Department of Medical Biology, Medical University of Bialystok, Mickiewicza 2C, 15-222 Bialystok, Poland; e-mail: piotr.zabielski@umb.edu.pl

***** Correspondence: Jerzy Palka, e-mail: pal@umb.edu.pl (J.P.)

1. Results

*1.1.* *Efficacy of shRNA-based PRODH/POX knock-down in MCF-7 cells*

*
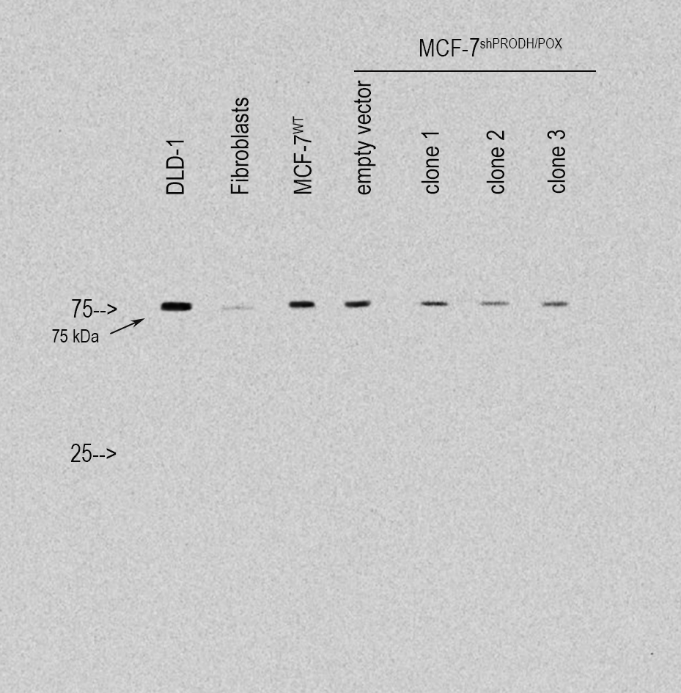
*

*
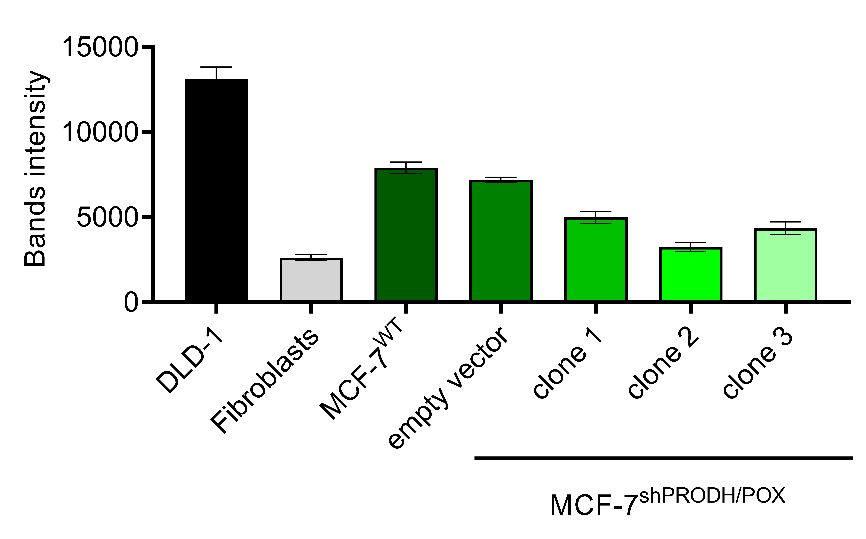
*

SFig. 1. Expression of PRODH/POX (POX) in MCF-7^WT^ and MCF-7^shPRODH/POX^ cells. Transfection of the MCF-7cells with different PRODH/POX shRNA constructs (clone 1-3) were done. DLD-1 cells were use.d as a positive control and fibroblasts as a negative control for the expression of PRODH/POX. Representative gels of Western blotting and the intensity of POX bands was quantified by densitometry and normalized to β-actin, values represent the mean (% of control) ± SD of three experiments, *P <0.001.

*1.2. Cell viability and DNA biosynthesis in MCF-7 PRODH/POX knock-down cells (MCF-7^shPRODH/POX^) versus MCF-7^WT^ cells.*

*
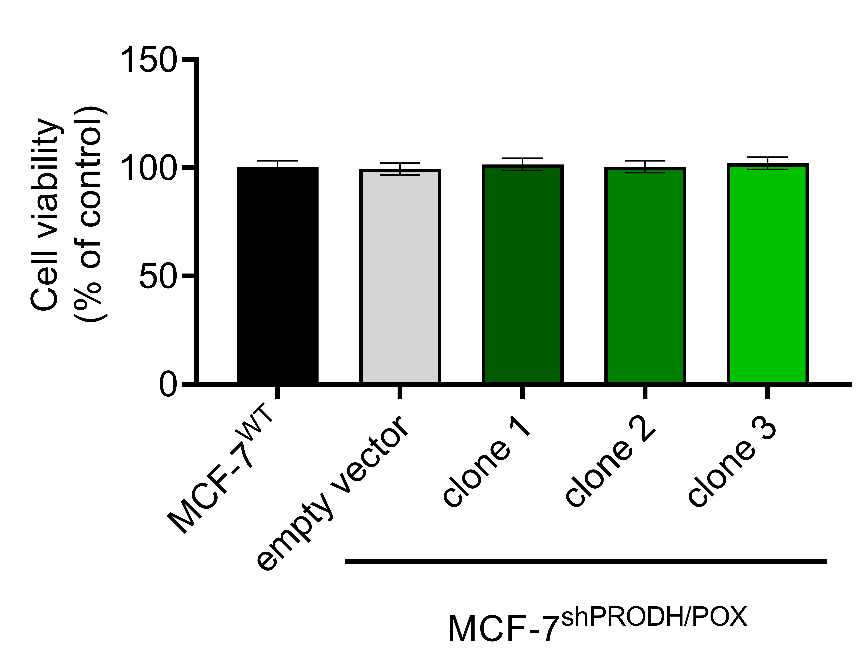
*

SFig. 2. Cell viability in MCF-7^WT^ and clones of PRODH/POX silenced MCF-7 (*MCF-7^shPRODH/POX^)* cells. The mean values ± SD from 3 experiments done in duplicates are presented, *P <0.001.

*
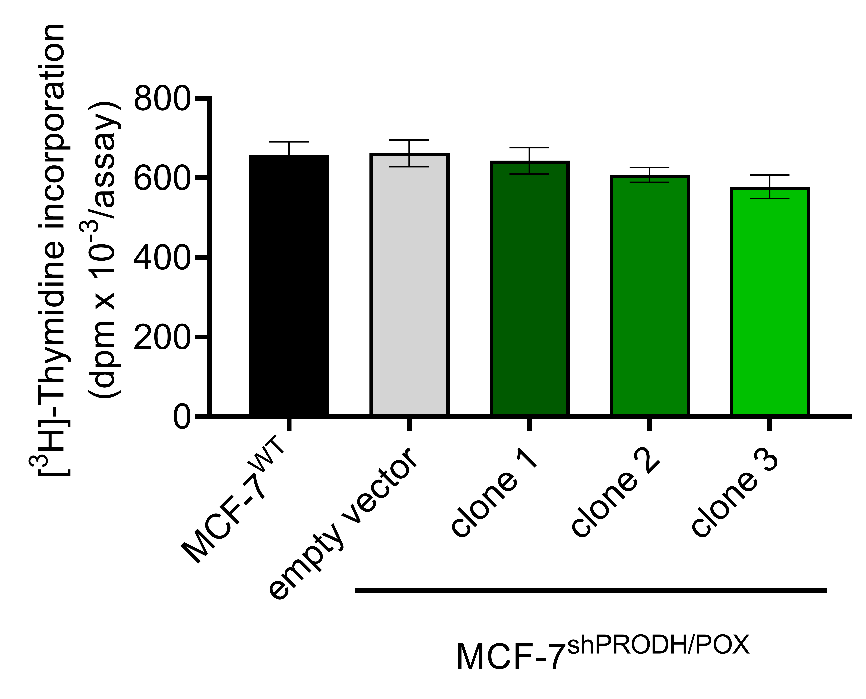
*

SFig. 3. DNA biosynthesis in MCF-7^WT^ and clones of PRODH/POX silenced MCF-7 (*MCF-7^shPRODH/POX^)* cells. The mean values ± SD from 3 experiments done in duplicates are presented, *P <0.001.

*1.3. The effect of proline availability on cell viability and DNA biosynthesis in MCF-7^WT^ and MCF-7 PRODH/POX knock-down cells (MCF-7^iPOX^ cells)*

*
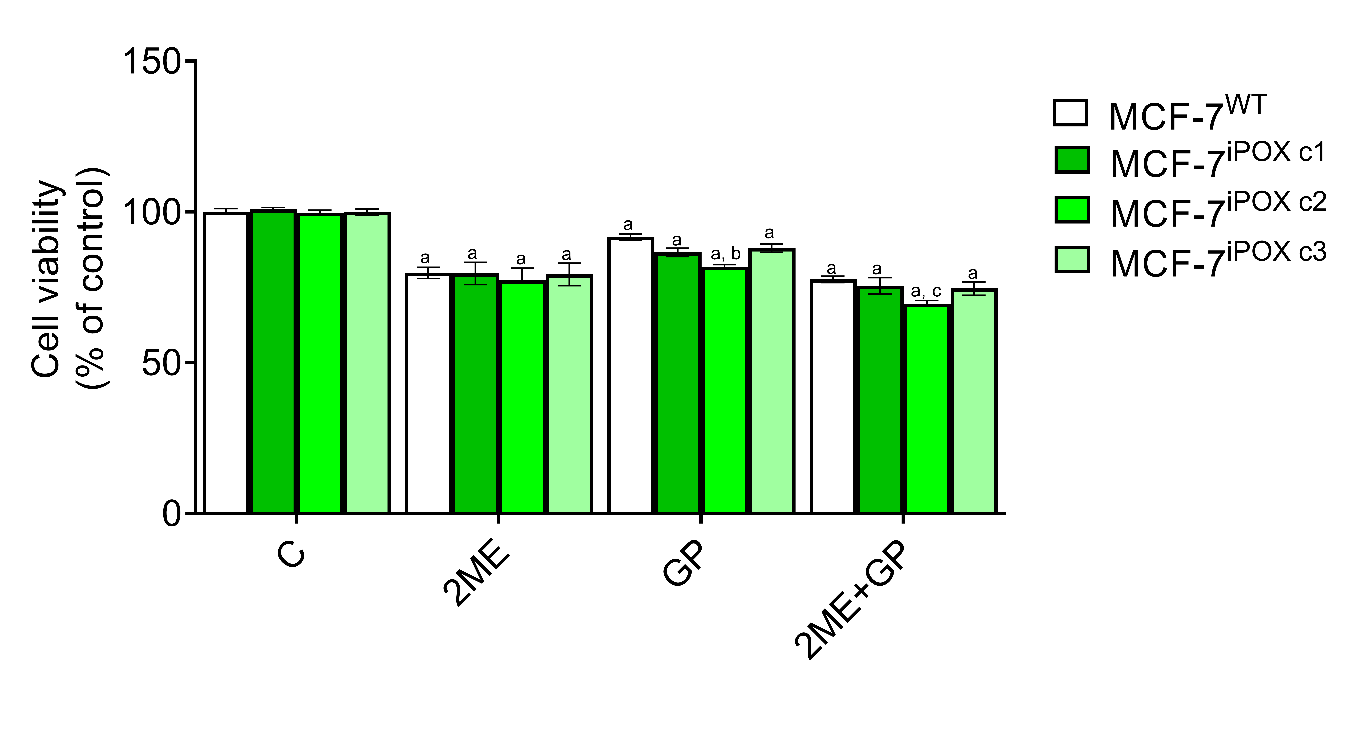
*

SFig. 4. Cell viability in MCF-7^WT^ and clones of PRODH/POX silenced MCF-7 (*MCF-7^shPRODH/POX^)* cells cultured in DMEM without glutamine with methoxyestradiol (2ME), glycyl-proline (GP) or 2ME+GP for 24 h. The mean values ± SD from 3 experiments done in duplicates are presented. Statistically significant differences (at the level <0.0001) are marked as: a vs. control MCF-7^WT^, b vs. MCF-7^WT^ treated by GP and c vs. MCF-7^WT^ treated by 2ME+GP.

*
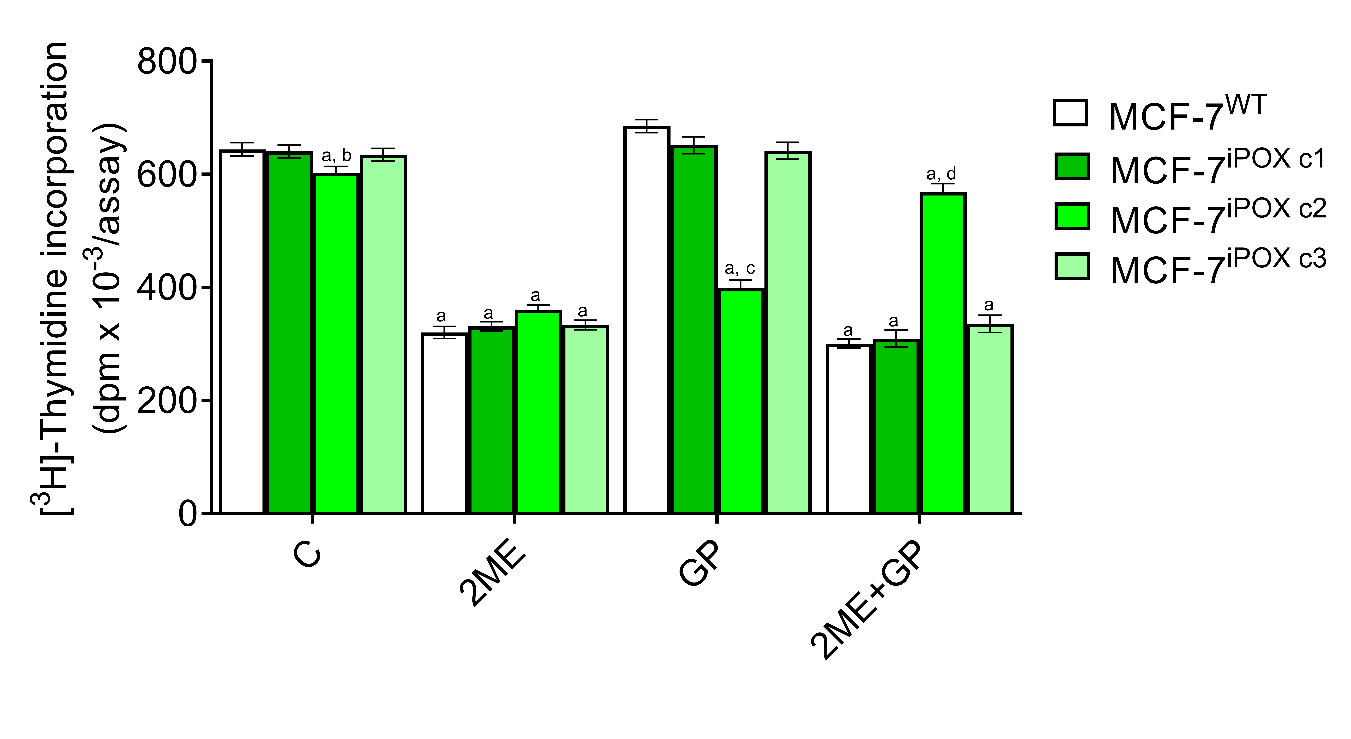
*

SFig. 5. DNA biosynthesis in MCF-7^WT^ and clones of PRODH/POX silenced MCF-7 (*MCF-7^shPRODH/POX^)* cells cultured in DMEM without glutamine with methoxyestradiol (2ME), glycyl-proline (GP) or 2ME+GP for 24 h. The mean values ± SD from 3 experiments done in duplicates are presented, *P <0.001. Statistically significant differences (at the level <0.0001) are marked as: a vs. control MCF-7^WT^, b vs. MCF-7^WT^ treated by 2ME, c vs. MCF-7^WT^ treated by GP and d vs. MCF-7^WT^ treated by 2ME+GP.

*1.4. Cell viability and DNA biosynthesis in MCF-7 PRODH/POX knock-down cells (MCF-7^iPOX^) versus MCF-7^WT^ cells treated methoxyestradiol (2ME), glycyl-proline (GP) and 2ME+GP for 24 h, 48 h and 72 h.*

*
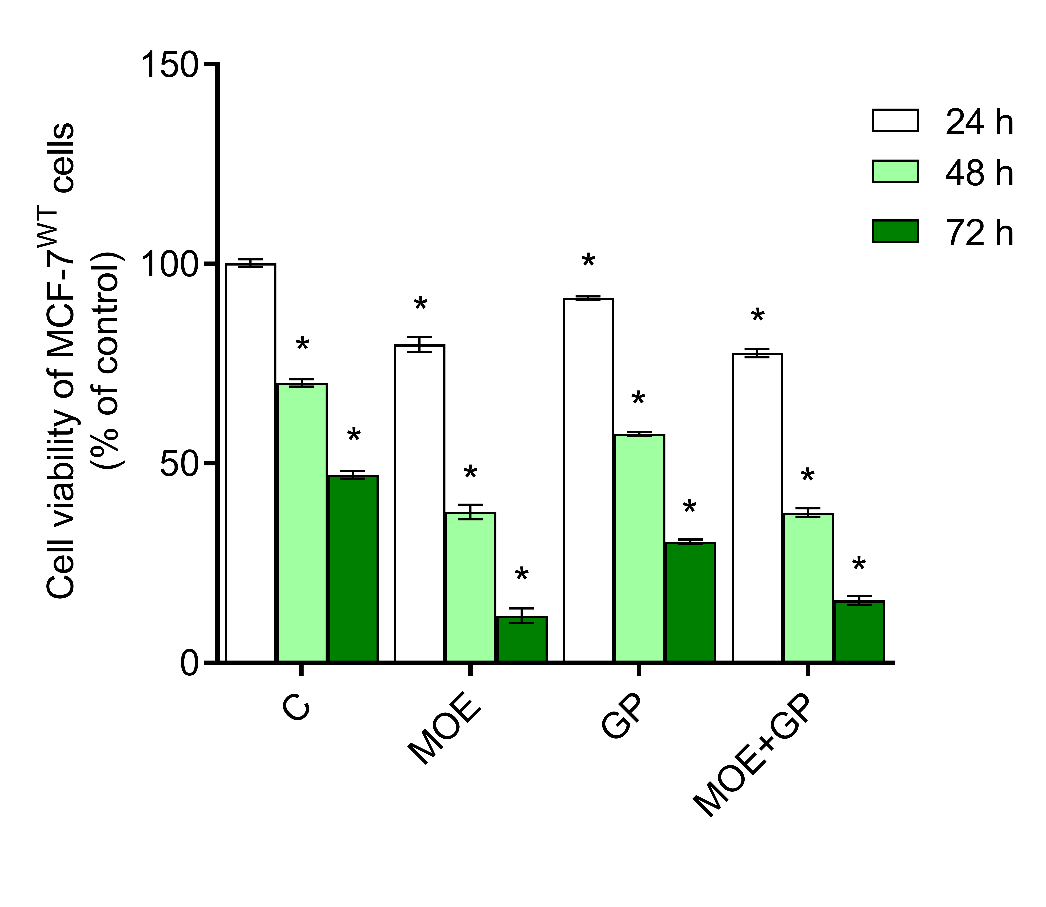
*

SFig. 6. Cell vitality in MCF-7^WT^ cells cultured in DMEM without glutamine with methoxyestradiol (2ME), glycyl-proline (GP) or 2ME+GP for 24 h, 48 h and 72 h. The mean values ± SD from 3 experiments done in duplicates are presented, *P <0.001. treated by 2ME+GP.


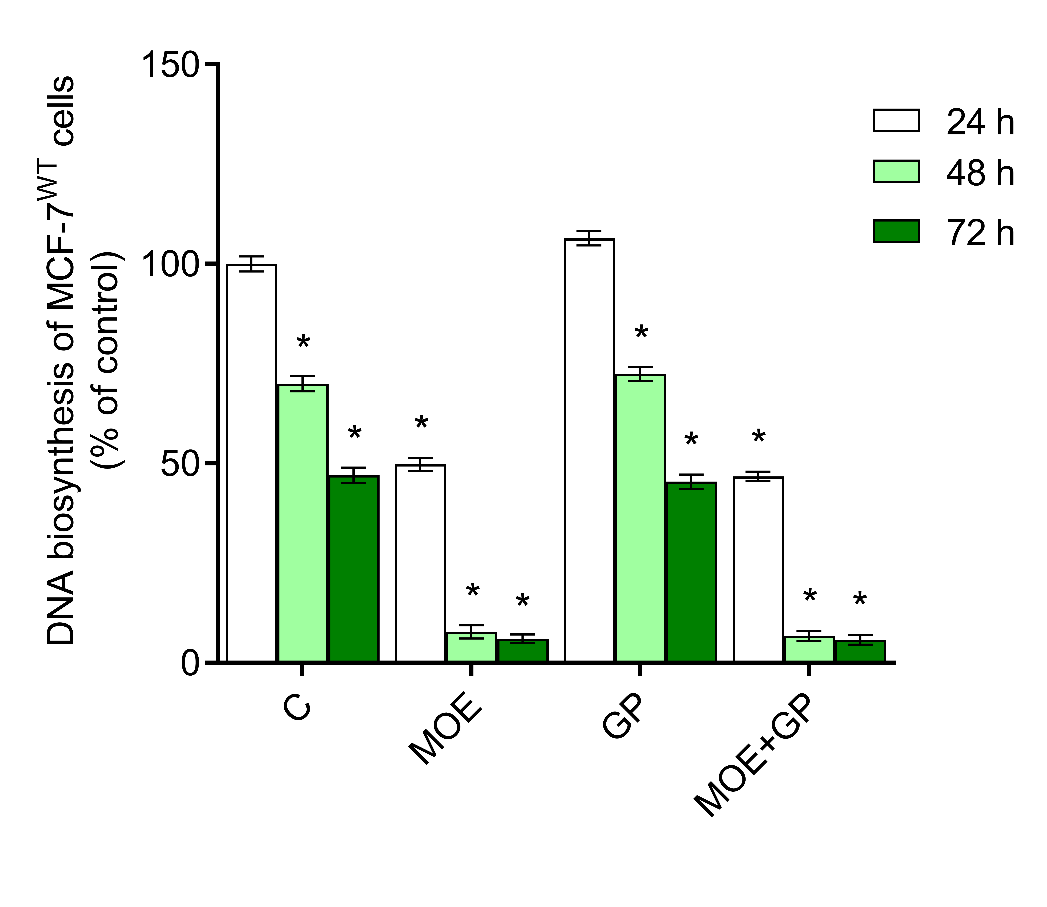


SFig. 7. DNA biosynthesis in MCF-7^WT^ cells cultured in DMEM without glutamine with methoxyestradiol (2ME), glycyl-proline (GP) or 2ME+GP for 24 h, 48 h and 72 h. The mean values ± SD from 3 experiments done in duplicates are presented, *P <0.001. treated by 2ME+GP.

*
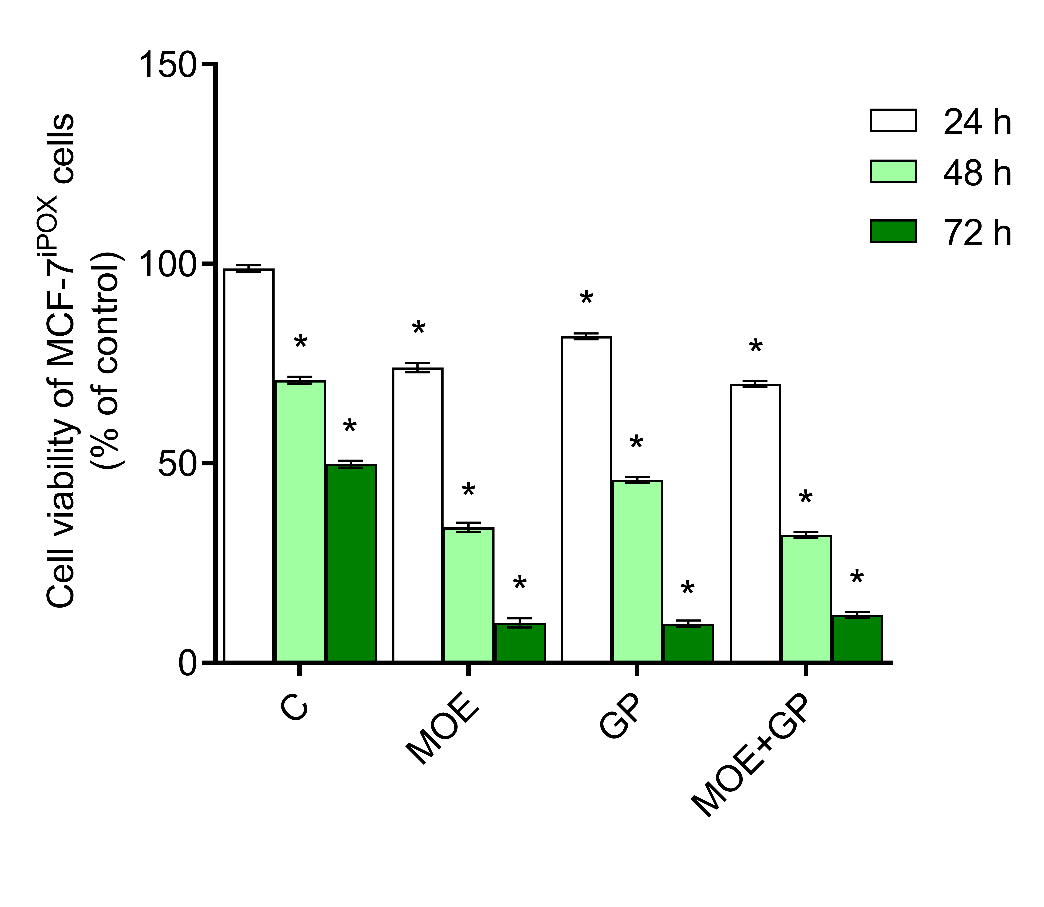
*

SFig. 8. Cell vitality in MCF-7^iPOX^ cells cultured in DMEM without glutamine with methoxyestradiol (2ME), glycyl-proline (GP) or 2ME+GP for 24 h, 48 h and 72 h. The mean values ± SD from 3 experiments done in duplicates are presented, *P <0.001. treated by 2ME+GP.

*
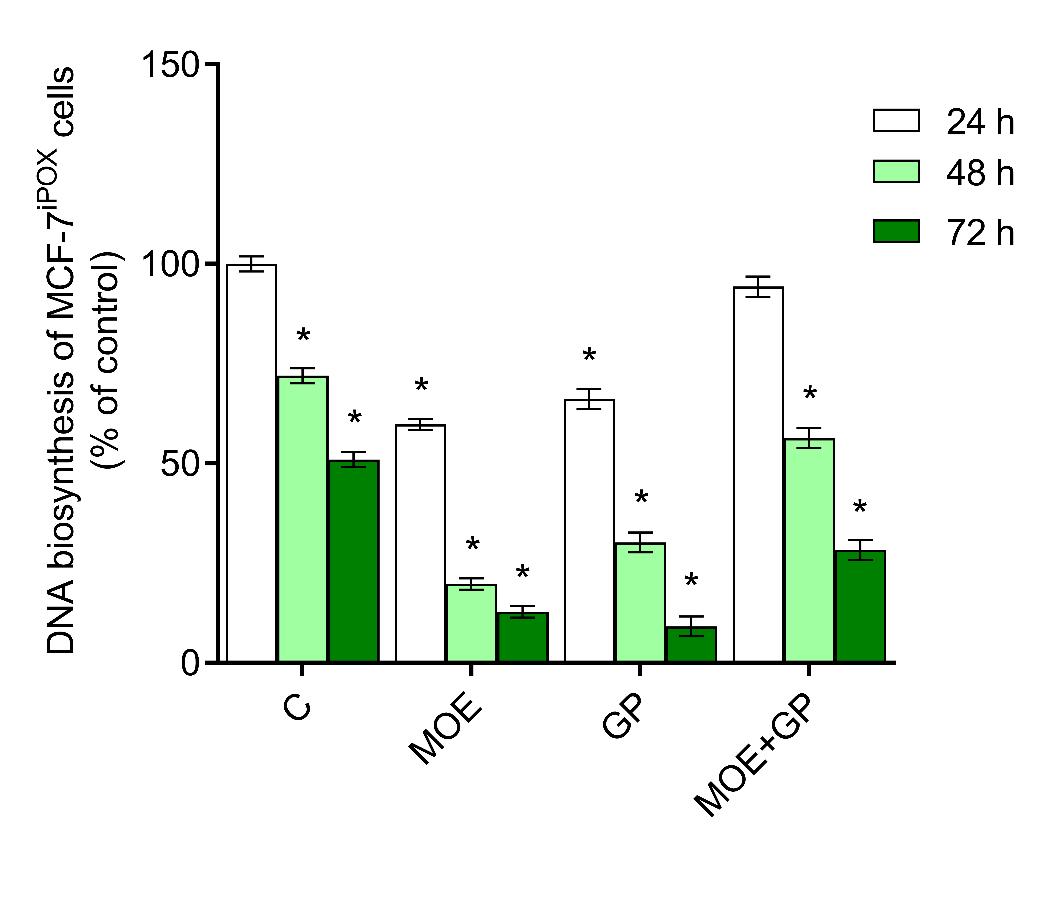
*

SFig. 9. DNA biosynthesis in MCF-7^iPOX^ cells cultured in DMEM without glutamine with methoxyestradiol (2ME), glycyl-proline (GP) or 2ME+GP for 24 h, 48 h and 72 h. The mean values ± SD from 3 experiments done in duplicates are presented, *P <0.001. treated by 2ME+GP.

*1.5. Western blot analysis - Down regulation of POX induces pro-survival phenotype through p53-prolidase complex formation in MCF-7 cells.*

1.5.1. POX


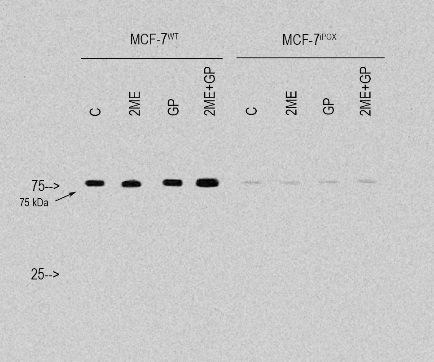

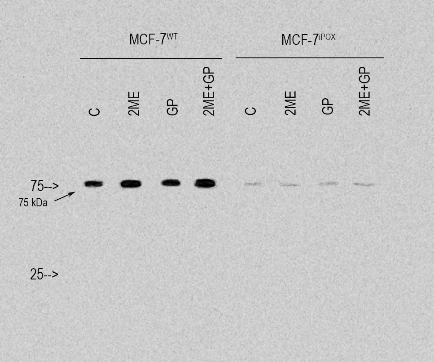

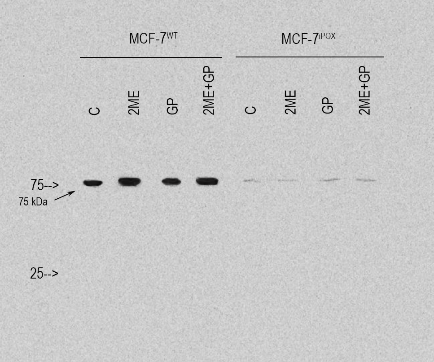


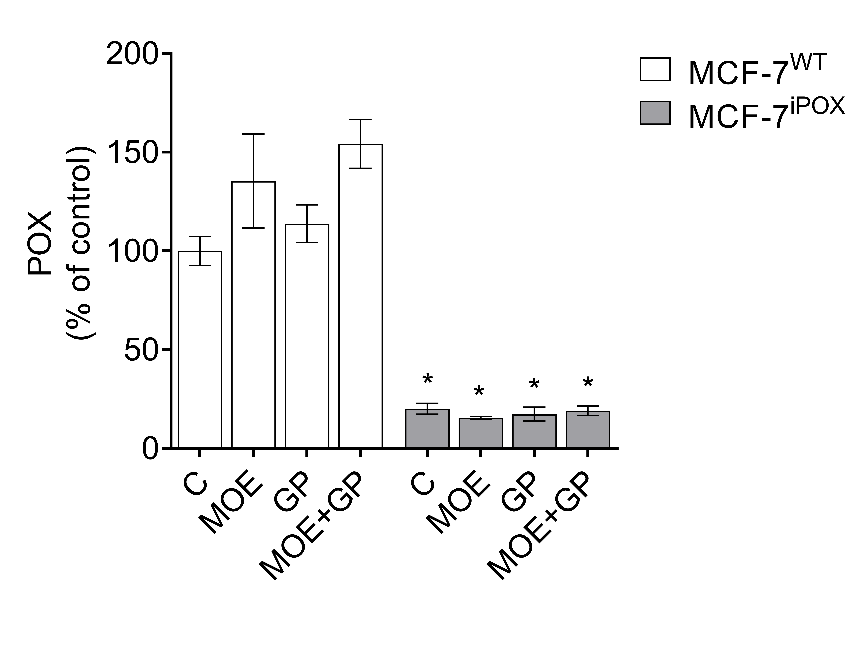


SFig. 10. The POX expression in MCF-7^WT^ cells and MCF-7^iPOX^ cells cultured in DMEM without glutamine and submitted for 24 h to methoxyestradiol (2ME), glycyl-proline (GP) or 2ME+GP. The WB bands intensity of representative gels was quantified by densitometry and normalized to β-actin. The densitometry values represent the mean (% of control) ± SD of three experiments, *P <0.001.

1.5.2. p53


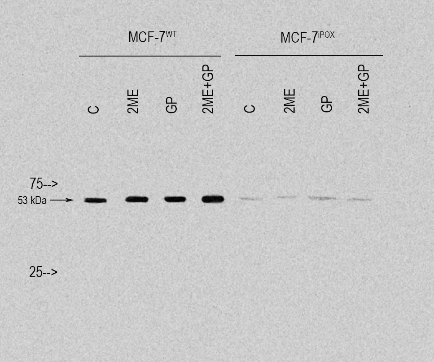

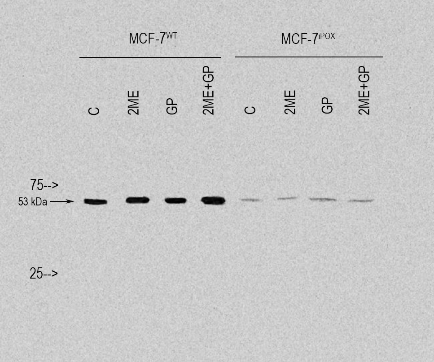

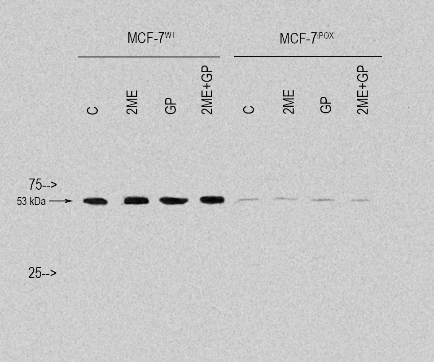


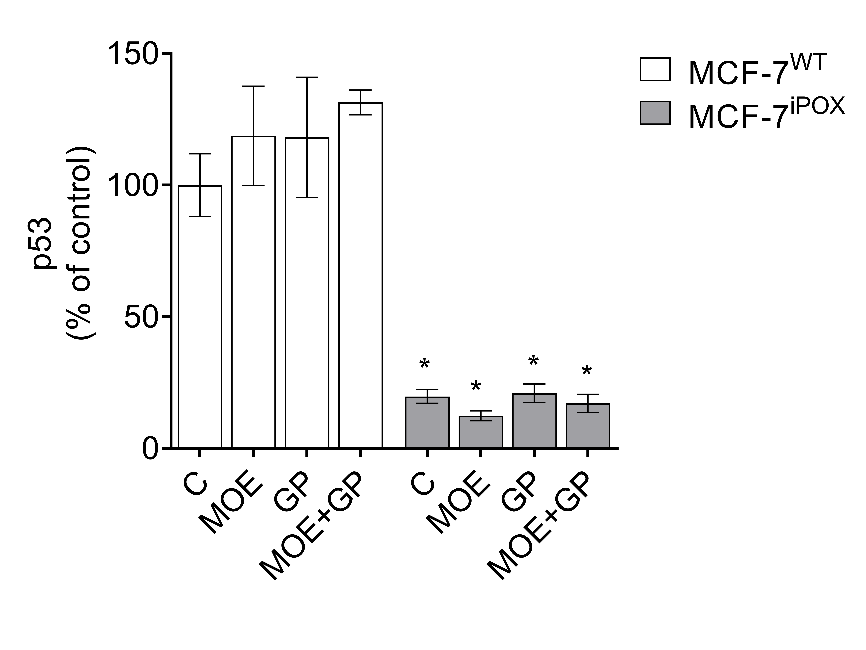


SFig. 11. The p53 expression in MCF-7^WT^ cells and MCF-7^iPOX^ cells cultured in DMEM without glutamine and submitted for 24 h to methoxyestradiol (2ME), glycyl-proline (GP) or 2ME+GP. The WB bands intensity of representative gels was quantified by densitometry and normalized to β-actin. The densitometry values represent the mean (% of control) ± SD of three experiments, *P <0.001.

1.5.3. cleaved-Caspase-9


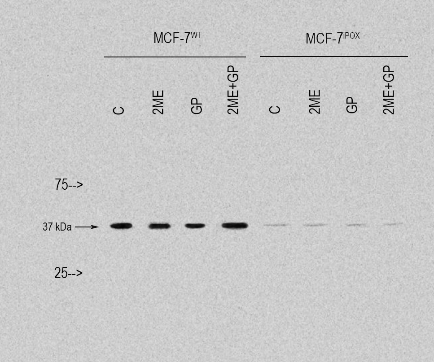

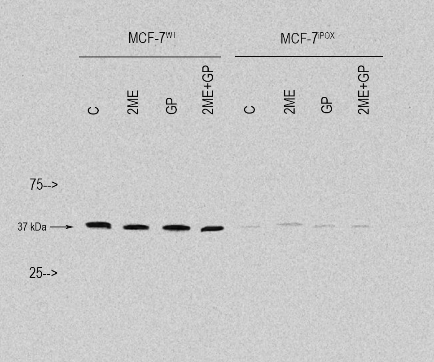

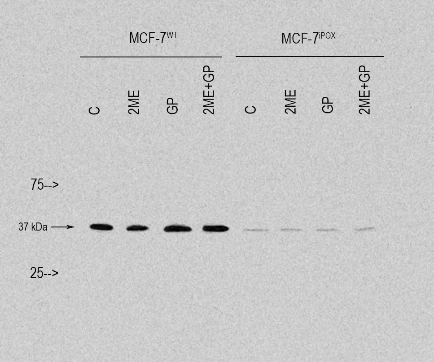


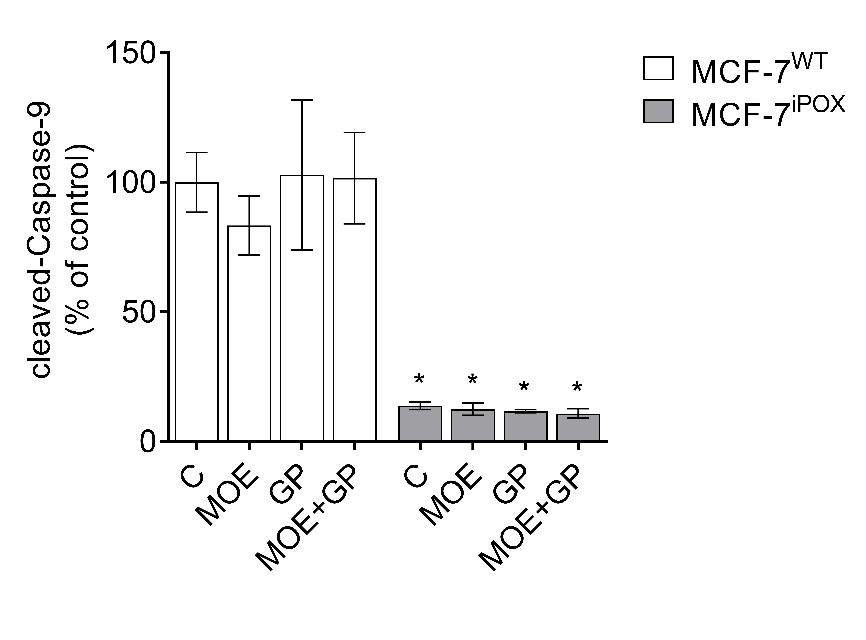


SFig.12. The cleaved-Caspase-9 expression in MCF-7^WT^ cells and MCF-7^iPOX^ cells cultured in DMEM without glutamine and submitted for 24 h to methoxyestradiol (2ME), glycyl-proline (GP) or 2ME+GP. The WB bands intensity of representative gels was quantified by densitometry and normalized to β-actin. The densitometry values represent the mean (% of control) ± SD of three experiments, *P <0.001.

1.5.4. Caspase-9


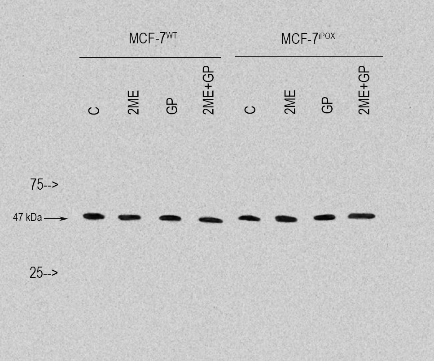

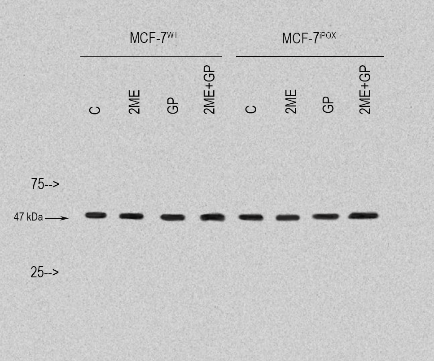

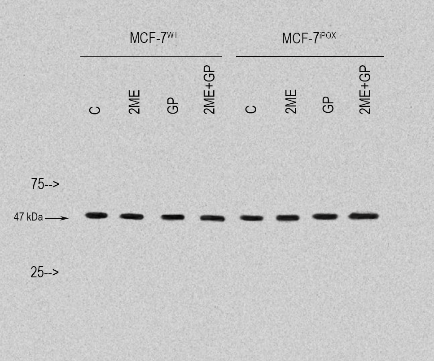


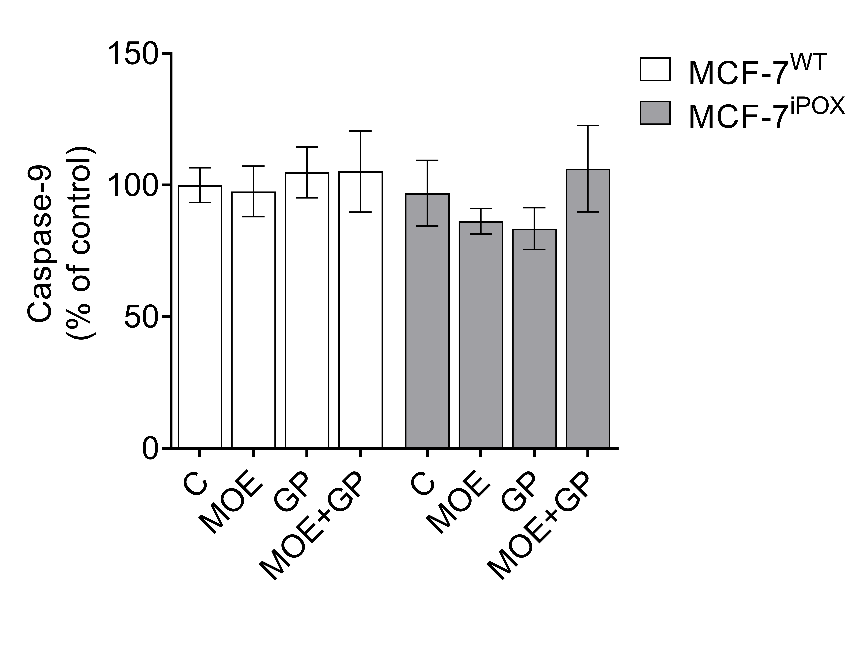


SFig. 13. The Caspase-9 expression in MCF-7^WT^ cells and MCF-7^iPOX^ cells cultured in DMEM without glutamine and submitted for 24 h to methoxyestradiol (2ME), glycyl-proline (GP) or 2ME+GP. The WB bands intensity of representative gels was quantified by densitometry and normalized to β-actin. The densitometry values represent the mean (% of control) ± SD of three experiments, *P <0.001.

1.5.5. cleaved-Caspase-3


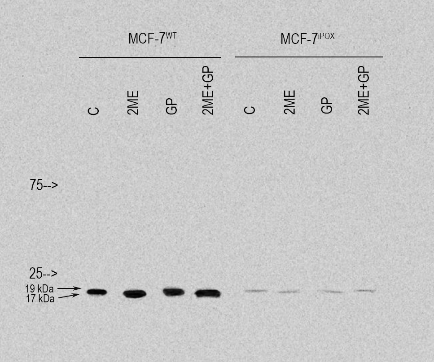

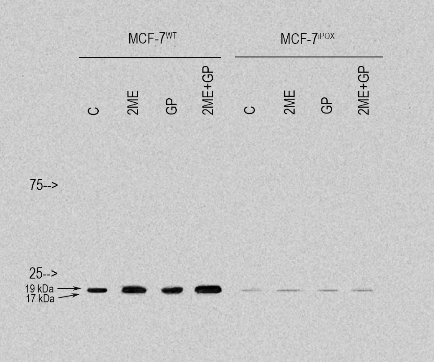

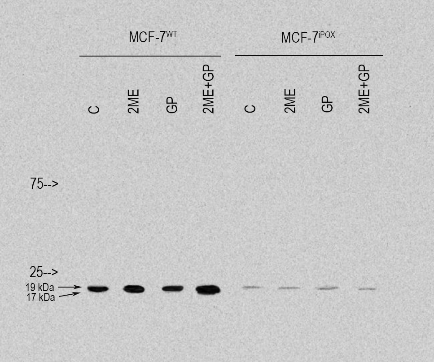


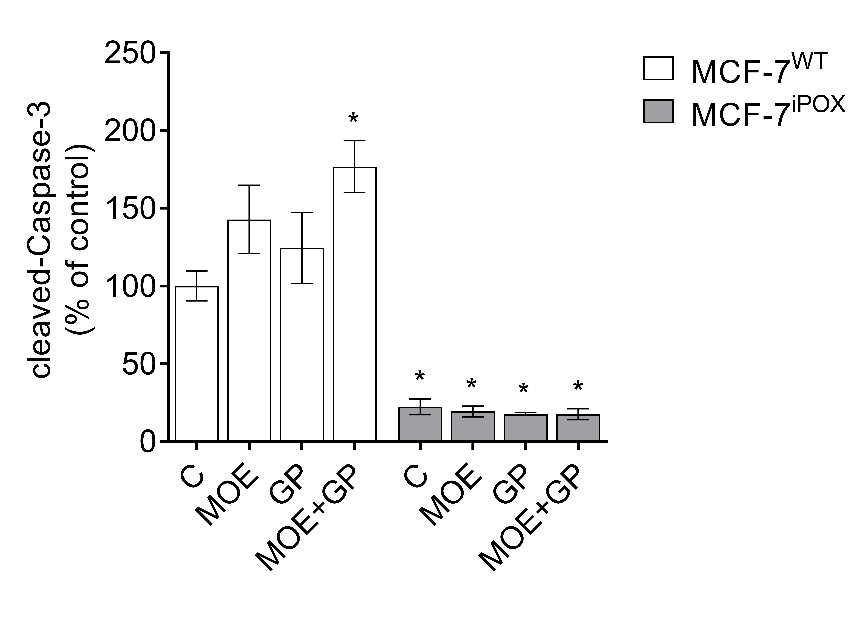


SFig. 14. The cleaved-Caspase-3 expression in MCF-7^WT^ cells and MCF-7^iPOX^ cells cultured in DMEM without glutamine and submitted for 24 h to methoxyestradiol (2ME), glycyl-proline (GP) or 2ME+GP. The WB bands intensity of representative gels was quantified by densitometry and normalized to β-actin. The densitometry values represent the mean (% of control) ± SD of three experiments, *P <0.001.

1.5.6. Caspase-3


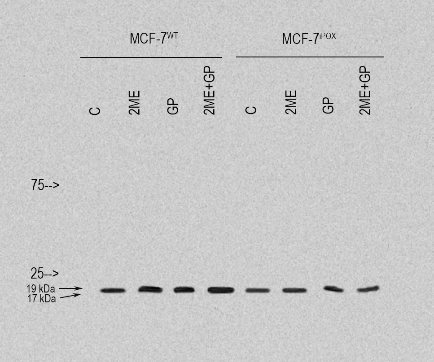

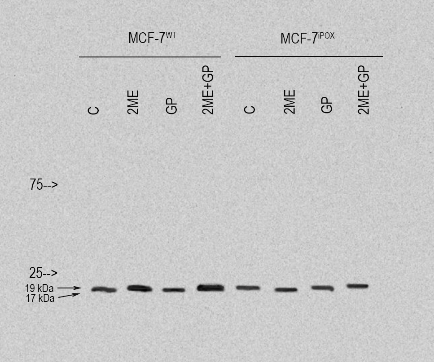

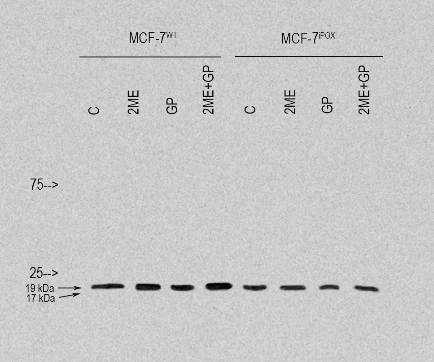


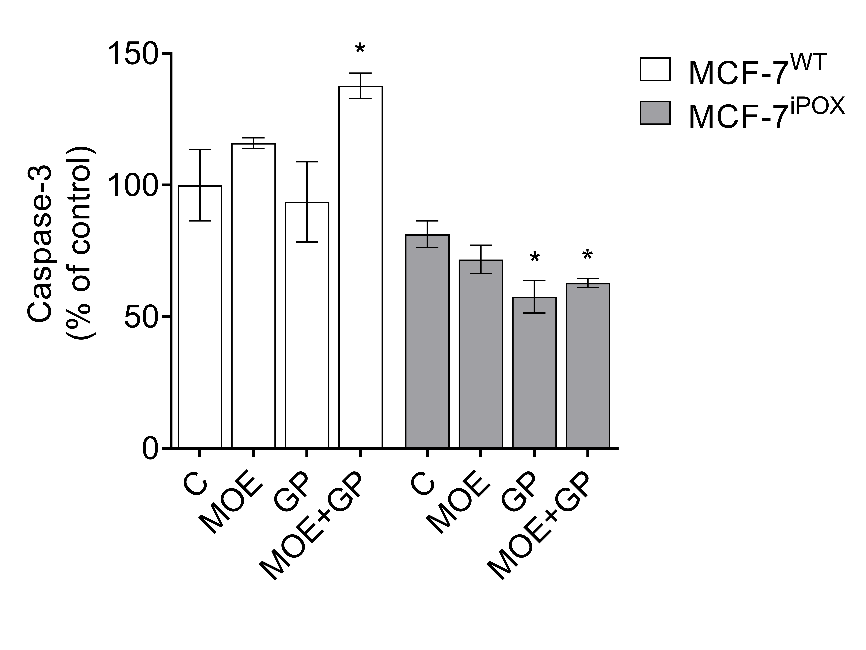


SFig. 15. The Caspase-3 expression in MCF-7^WT^ cells and MCF-7^iPOX^ cells cultured in DMEM without glutamine and submitted for 24 h to methoxyestradiol (2ME), glycyl-proline (GP) or 2ME+GP. The WB bands intensity of representative gels was quantified by densitometry and normalized to β-actin. The densitometry values represent the mean (% of control) ± SD of three experiments, *P <0.001.

1.5.7. Atg12


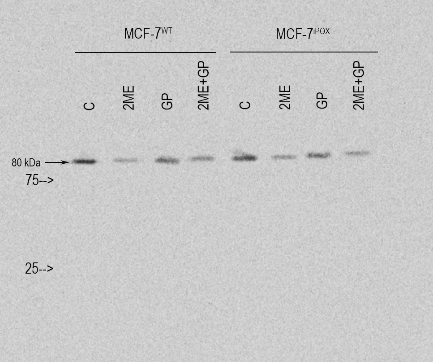

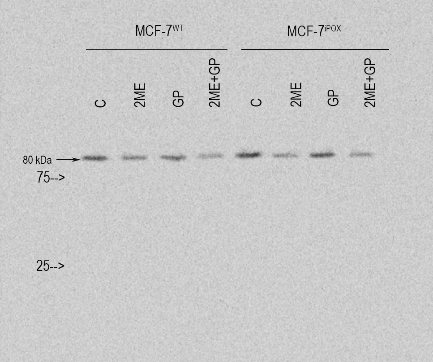

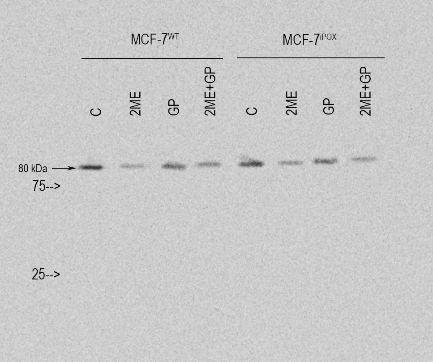


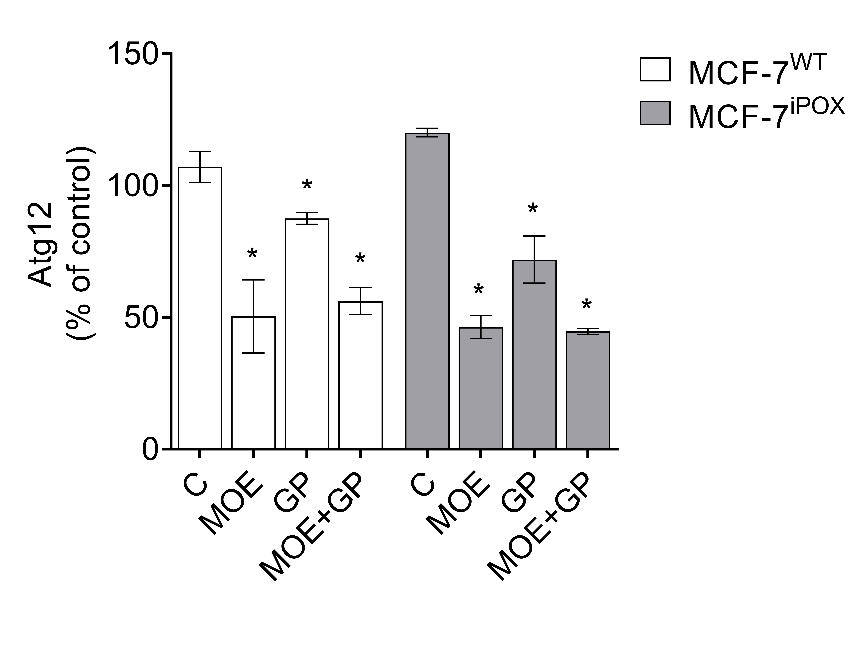


SFig. 16. The Atg12 expression in MCF-7^WT^ cells and MCF-7^iPOX^ cells cultured in DMEM without glutamine and submitted for 24 h to methoxyestradiol (2ME), glycyl-proline (GP) or 2ME+GP. The WB bands intensity of representative gels was quantified by densitometry and normalized to β-actin. The densitometry values represent the mean (% of control) ± SD of three experiments, *P <0.001.

1.5.8. LC3B


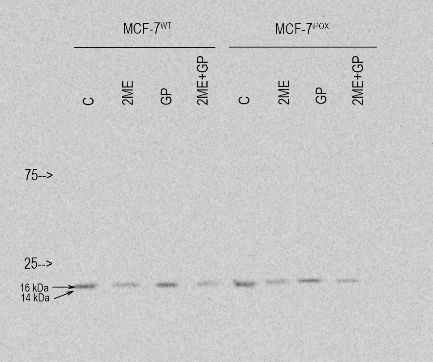

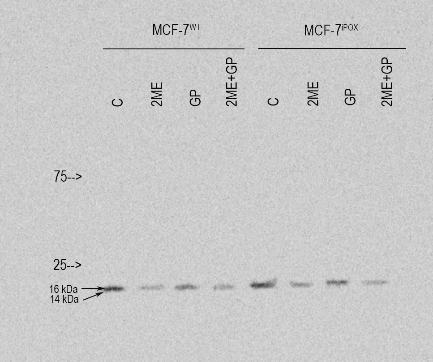

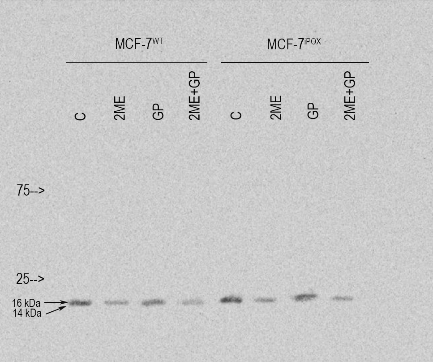


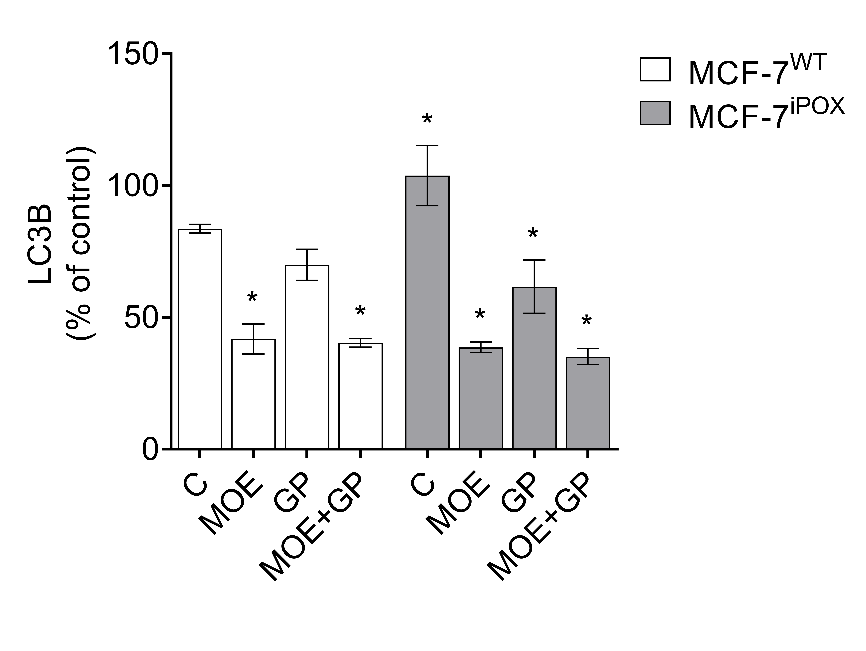


SFig. 17. The LC3B expression in MCF-7^WT^ cells and MCF-7^iPOX^ cells cultured in DMEM without glutamine and submitted for 24 h to methoxyestradiol (2ME), glycyl-proline (GP) or 2ME+GP. The WB bands intensity of representative gels was quantified by densitometry and normalized to β-actin. The densitometry values represent the mean (% of control) ± SD of three experiments, *P <0.001.

1.5.9.β-actin


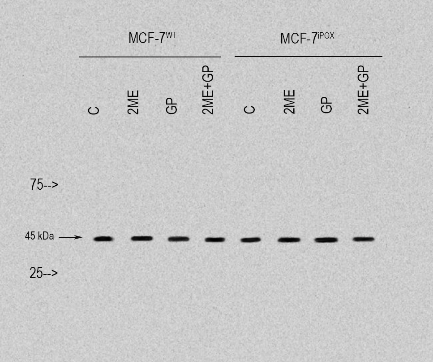

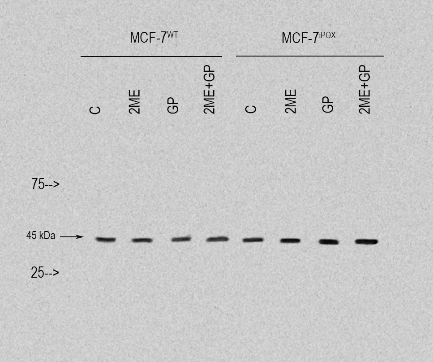

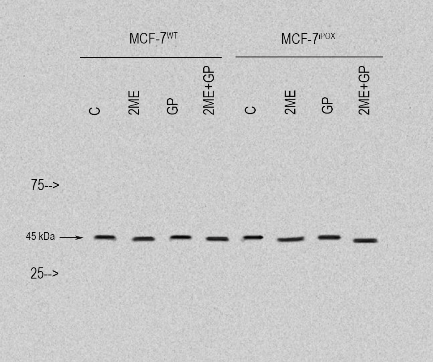


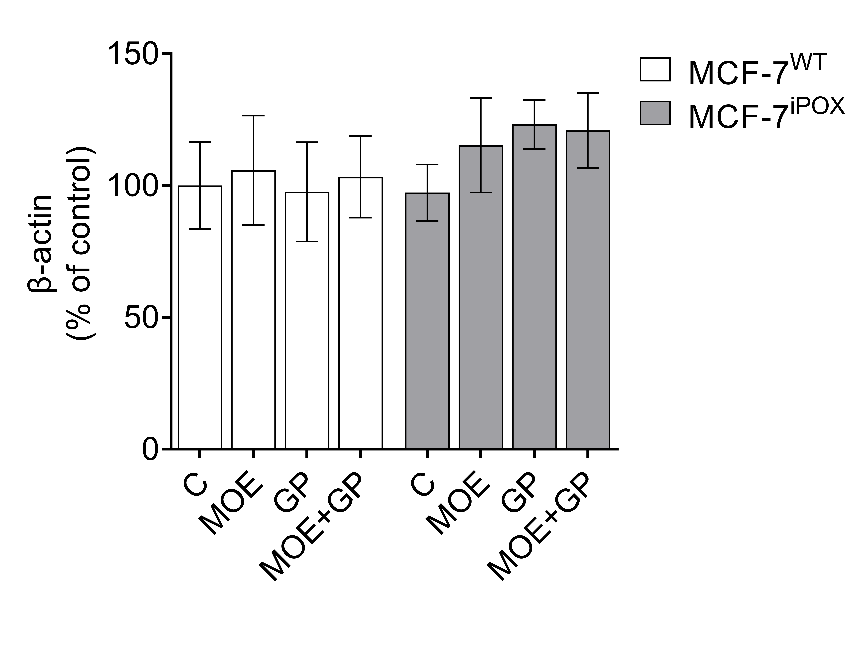


SFig. 18. β-actin expression in MCF-7^WT^ cells and MCF-7^iPOX^ cells cultured in DMEM without glutamine and submitted for 24 h to methoxyestradiol (2ME), glycyl-proline (GP) or 2ME+GP. Representative 3 of 18 gels of Western blotting and the intensity of β-actin bands was quantified by densitometry, values represent the mean (% of control) ± SD of three experiments, *P <0.001.

*1.6. PEPD Immunoprecipitation*

1.6.1. PEPD


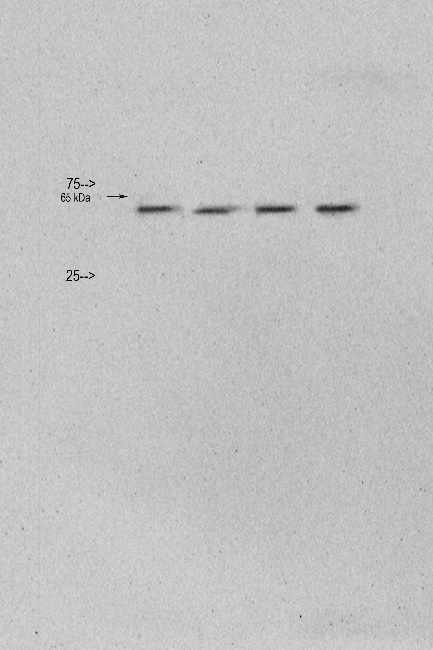

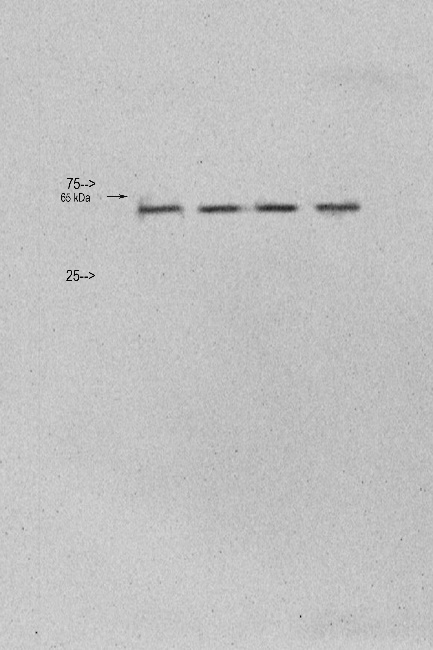

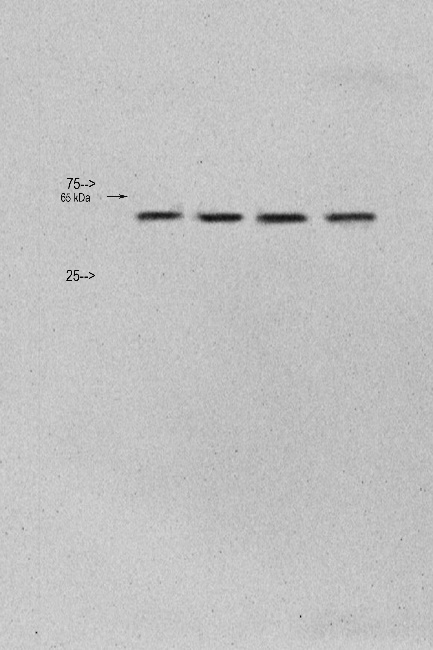


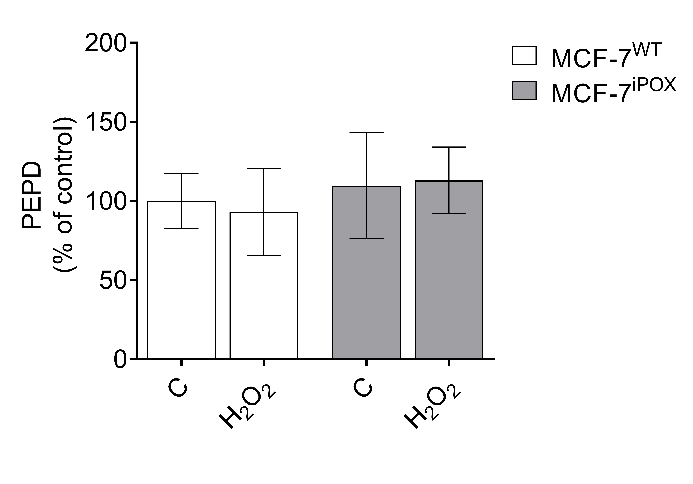


SFig. 19. The PEPD expression in lysates of PEPD immunoprecipitant coming from MCF-7^WT^ cells and MCF-7^iPOX^ cells in DMEM without glutamine and treated for 24 h with or without 400 µM hydrogen peroxide. The WB bands intensity of representative gels was quantified by densitometry and normalized to β-actin. The densitometry values represent the mean (% of control) ± SD of three experiments, *P <0.001.

1.6.2. IgG


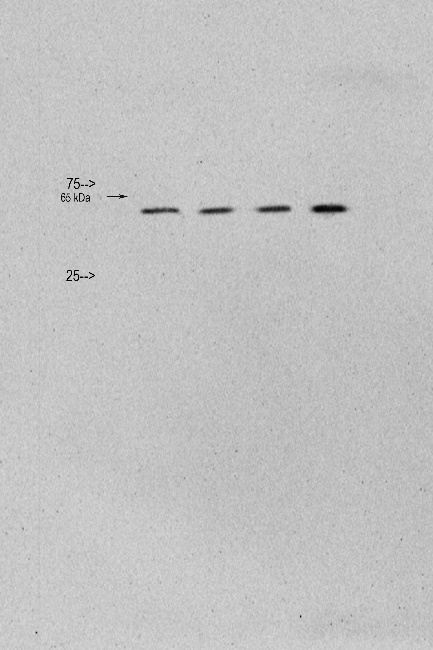

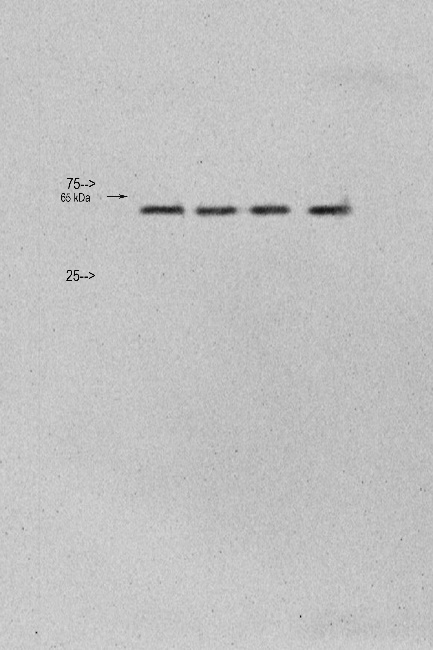

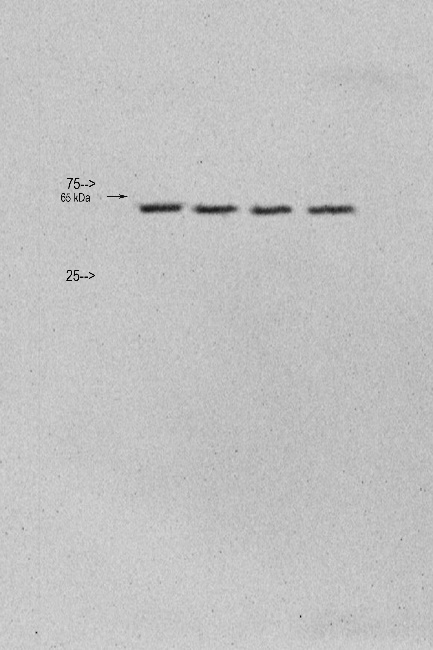


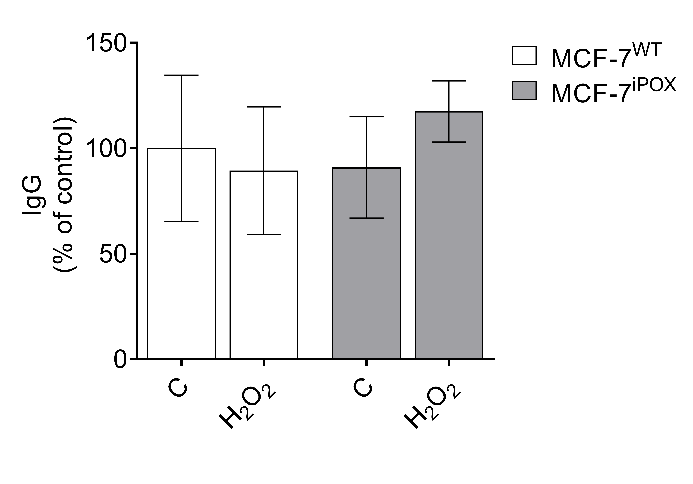


SFig. 20. The IgG expression in lysates of PEPD immunoprecipitant coming from MCF-7^WT^ cells and MCF-7^iPOX^ cells in DMEM without glutamine and treated for 24 h with or without 400 µM hydrogen peroxide. The WB bands intensity of representative gels was quantified by densitometry and normalized to β-actin. The densitometry values represent the mean (% of control) ± SD of three experiments, *P <0.001.

1.6.3. P53


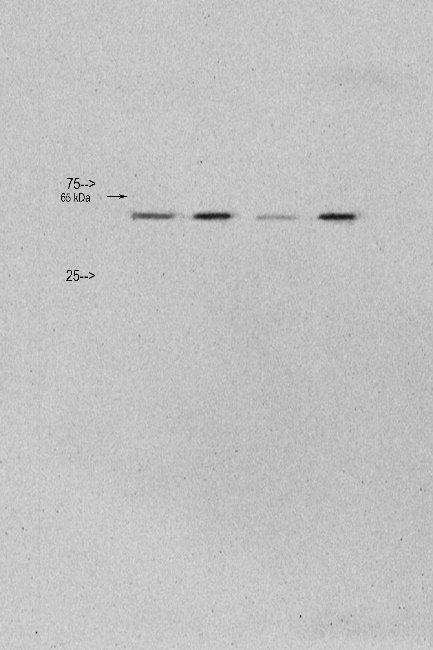

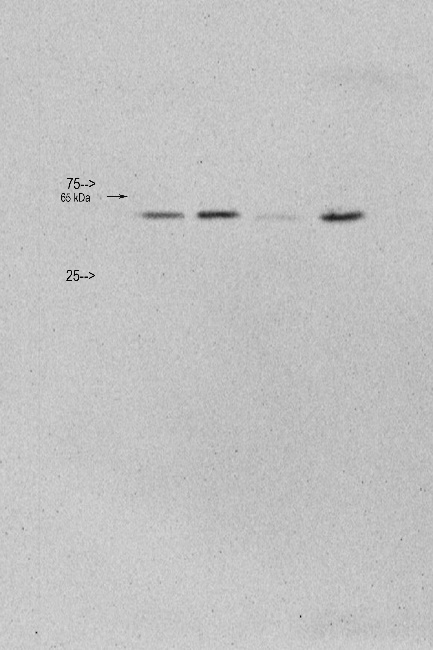

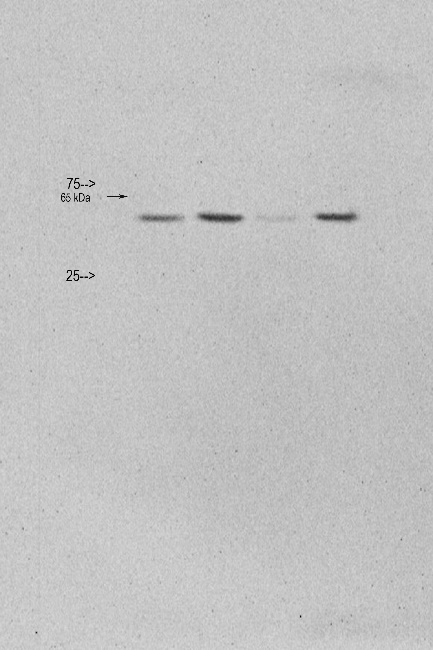


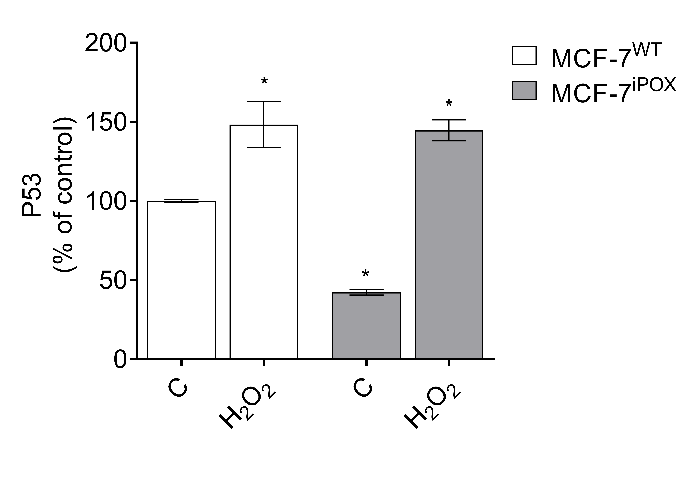


SFig. 21. The P53 expression in supernatant of PEPD immunoprecipitant coming from MCF-7^WT^ cells and MCF-7^iPOX^ cells in DMEM without glutamine and treated for 24 h with or without 400 µM hydrogen peroxide. The WB bands intensity of representative gels was quantified by densitometry and normalized to β-actin. The densitometry values represent the mean (% of control) ± SD of three experiments, *P <0.001.

1.6.4. POX


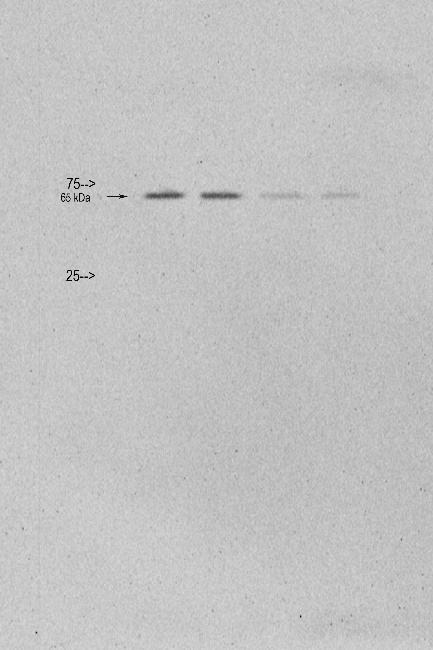

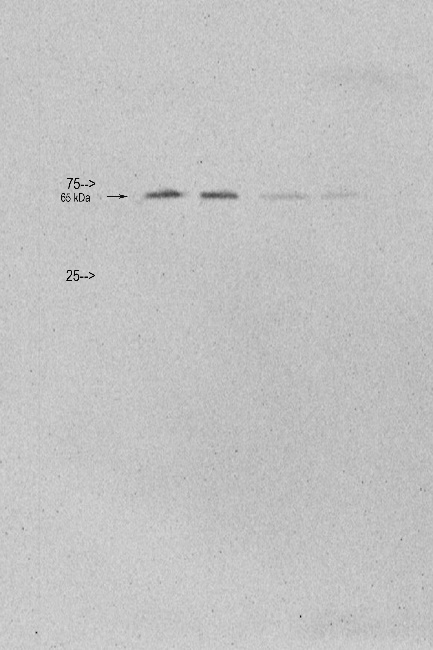

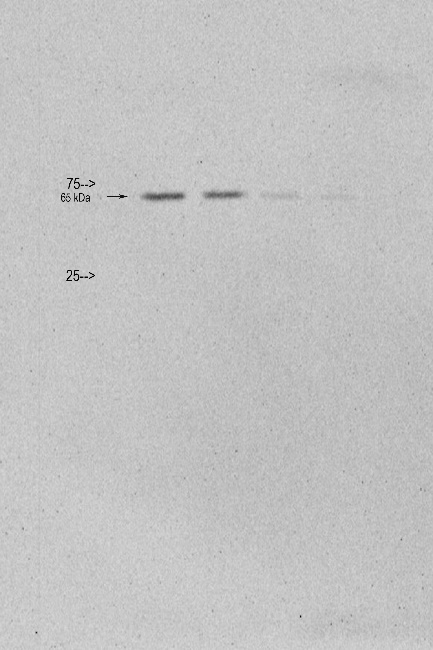


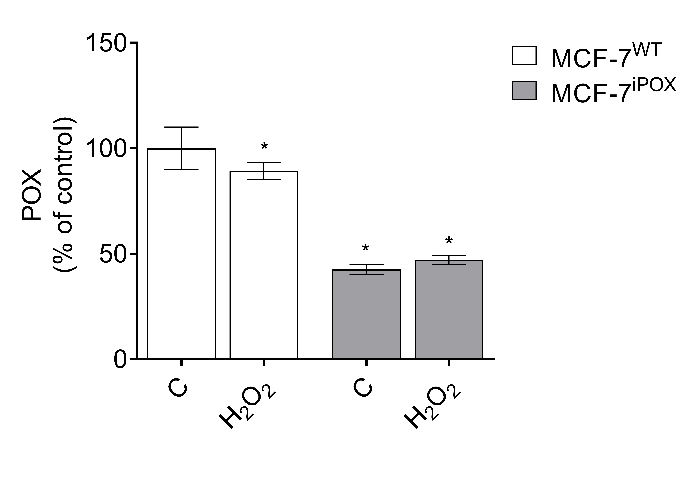


SFig. 22. The POX expression in supernatant of PEPD immunoprecipitant coming from MCF-7^WT^ cells and MCF-7^iPOX^ cells in DMEM without glutamine and treated for 24 h with or without 400 µM hydrogen peroxide. The WB bands intensity of representative gels was quantified by densitometry and normalized to β-actin. The densitometry values represent the mean (% of control) ± SD of three experiments, *P <0.001.

*1.7. P53 Immunoprecipitation*

1.7.1. P53


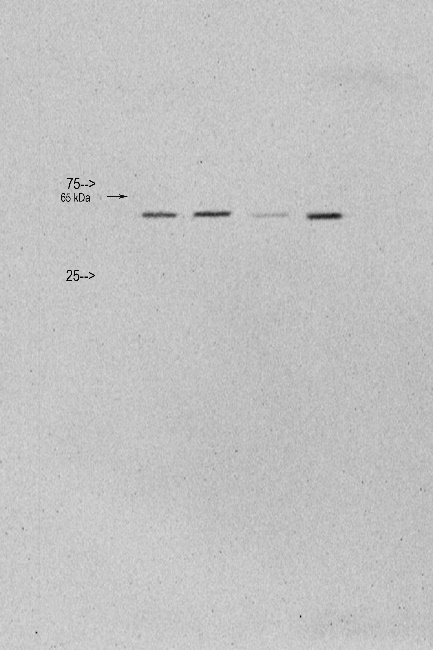

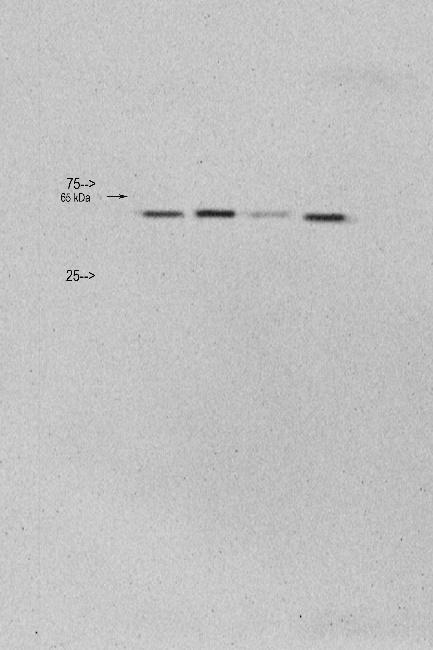

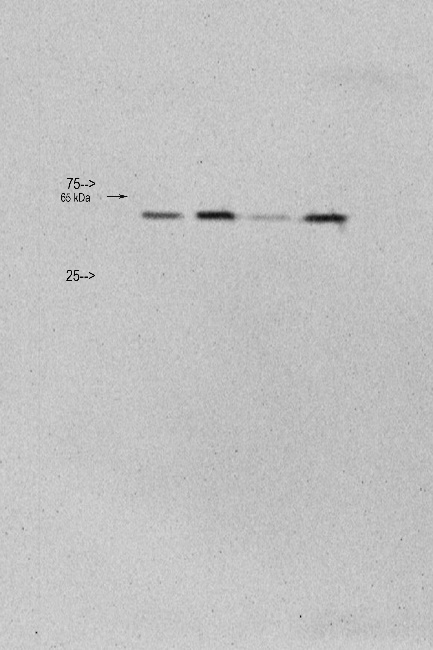


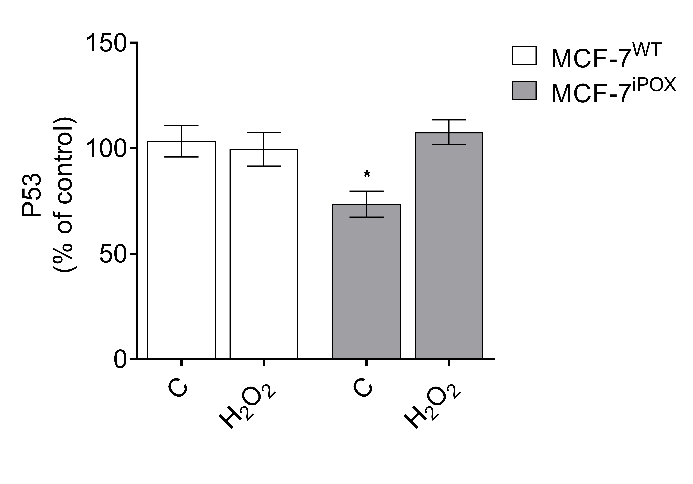


SFig. 23. The P53 expression in lysates of P53 immunoprecipitant coming from MCF-7^WT^ cells and MCF-7^iPOX^ cells in DMEM without glutamine and treated for 24 h with or without 400 µM hydrogen peroxide. The WB bands intensity of representative gels was quantified by densitometry and normalized to β-actin. The densitometry values represent the mean (% of control) ± SD of three experiments, *P <0.001.

1.7.2. IgG


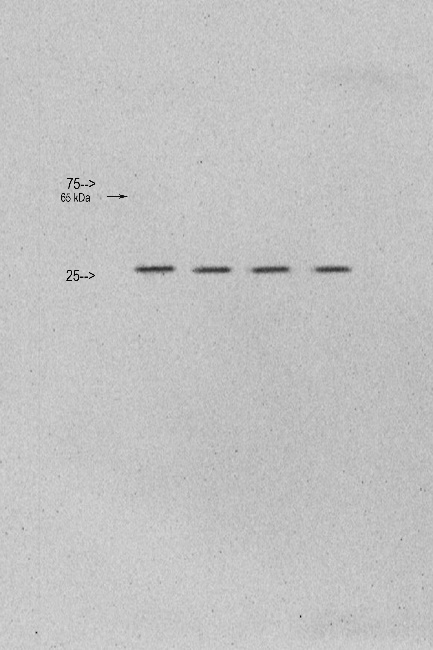

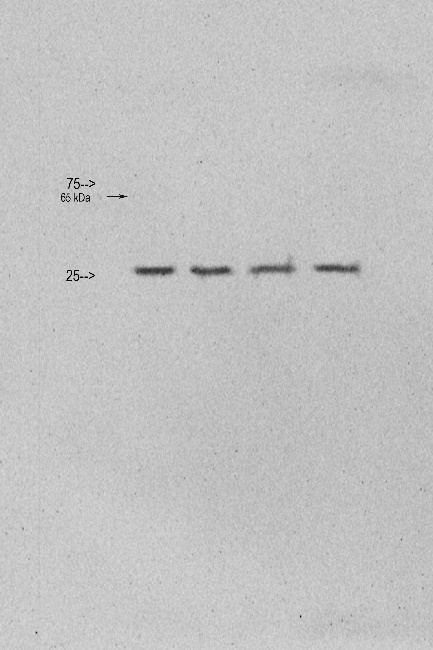

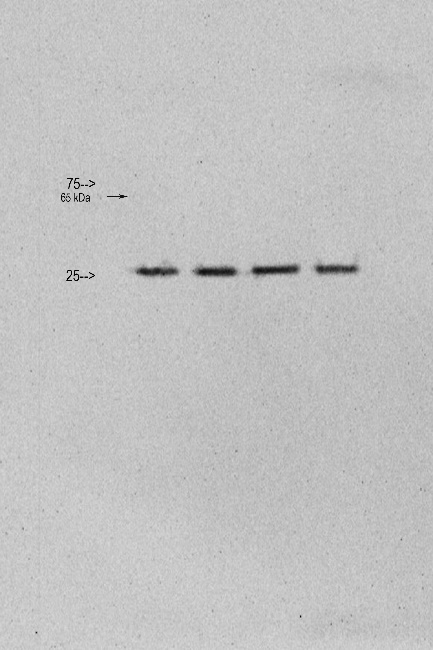


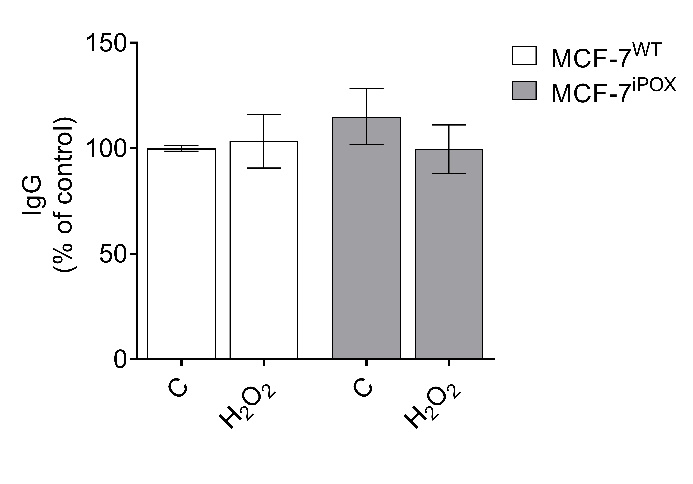


SFig. 24. The IgG expression in lysates of P53 immunoprecipitant coming from MCF-7^WT^ cells and MCF-7^iPOX^ cells in DMEM without glutamine and treated for 24 h with or without 400 µM hydrogen peroxide. The WB bands intensity of representative gels was quantified by densitometry and normalized to β-actin. The densitometry values represent the mean (% of control) ± SD of three experiments, *P <0.001.

1.7.3. PEPD


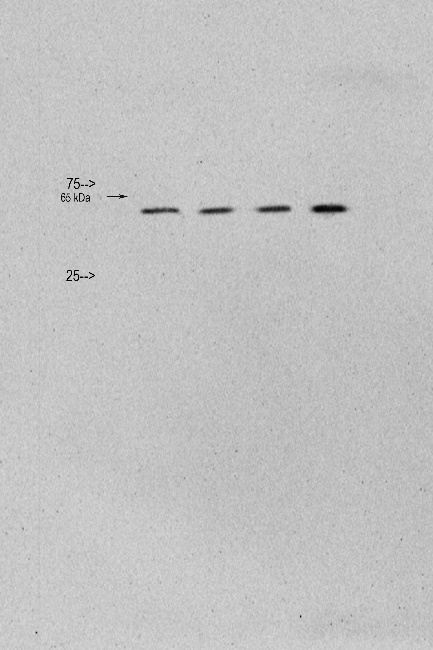

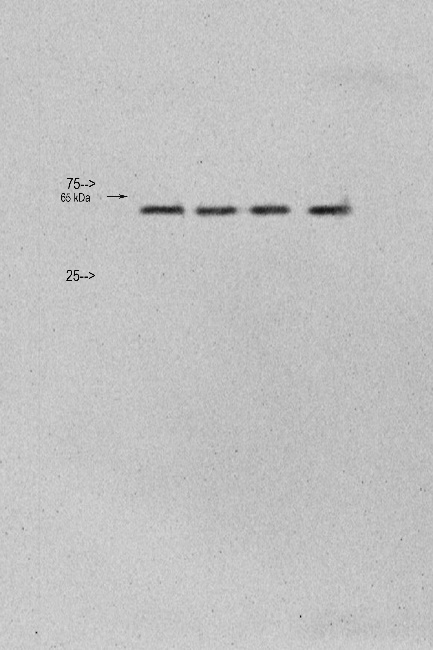

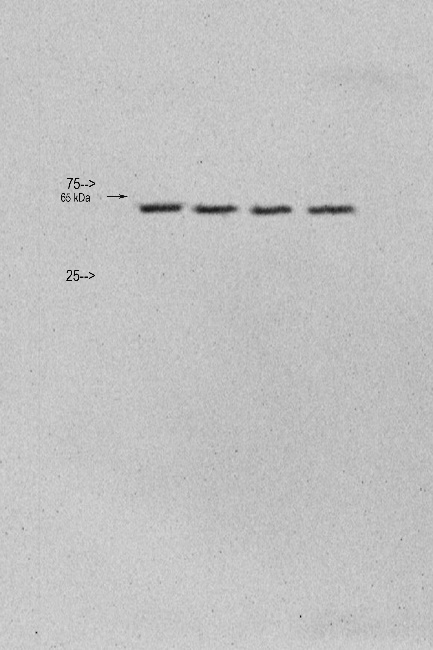


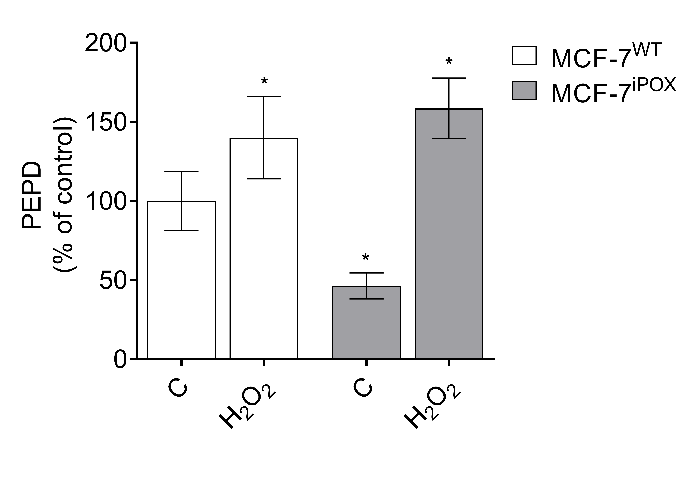


SFig. 25. The PEPD expression in supernatants of P53 immunoprecipitant coming from MCF-7^WT^ cells and MCF-7^iPOX^ cells in DMEM without glutamine and treated for 24 h with or without 400 µM hydrogen peroxide. The WB bands intensity of representative gels was quantified by densitometry and normalized to β-actin. The densitometry values represent the mean (% of control) ± SD of three experiments, *P <0.001.

1.7.4. POX


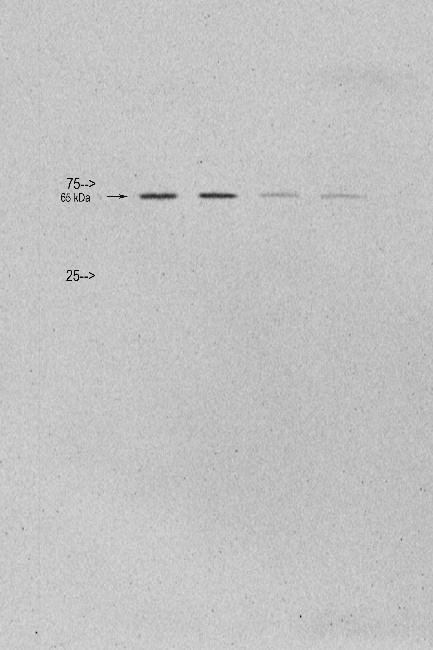

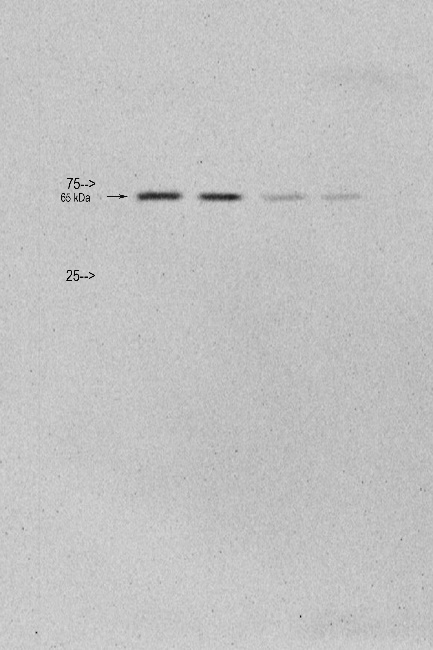

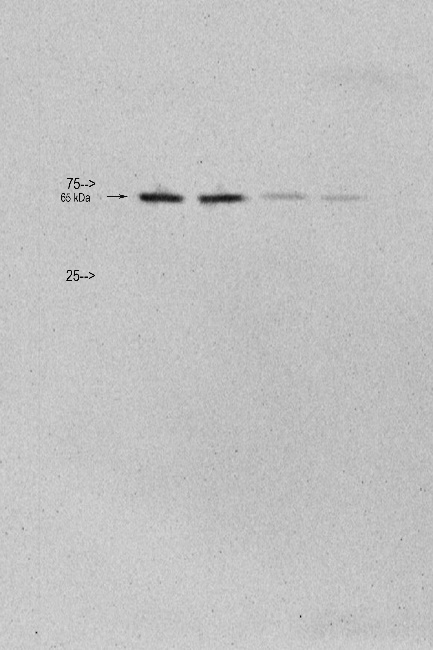


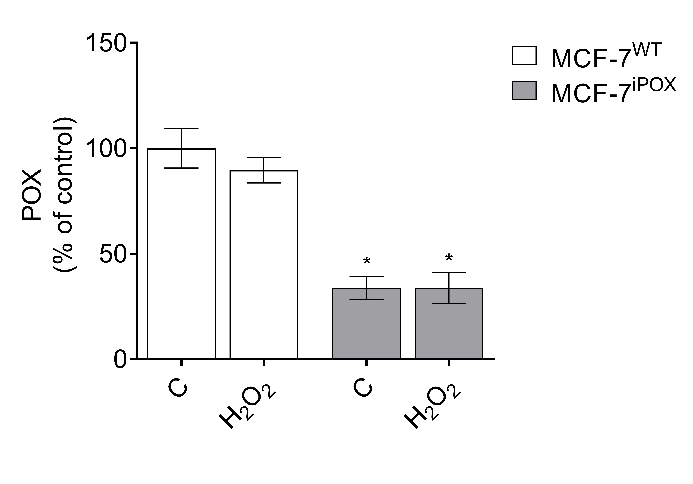


SFig. 26. The POX expression in supernatantss of P53 immunoprecipitant coming from MCF-7^WT^ cells and MCF-7^iPOX^ cells in DMEM without glutamine and treated for 24 h with or without 400 µM hydrogen peroxide. The WB bands intensity of representative gels was quantified by densitometry and normalized to β-actin. The densitometry values represent the mean (% of control) ± SD of three experiments, *P <0.001.
